# Diagnosis and Treatment of Primary Inflammatory Choriocapillaropathies (PICCPs): A Comprehensive Overview

**DOI:** 10.3390/medicina58020165

**Published:** 2022-01-21

**Authors:** Ioannis Papasavvas, Carl P. Herbort

**Affiliations:** Retinal and Inflammatory Eye Diseases, Centre for Ophthalmic Specialized Care (COS), 1003 Lausanne, Switzerland

**Keywords:** PICPPS, MEWDS, APMPPE, MFC, SC, ICGA, FA, non-infectious posterior uveitis, OCT-A, SDOCT

## Abstract

Purpose: Primary inflammatory choriocapillaropathies (PICCPs) belong to a group of intraocular inflammatory diseases with the common characteristic of inflammatory choriocapillaris hypo- or non-perfusion as the main clinicopathological mechanism. The purpose of our article is to describe clinical characteristics and multimodal imaging, that can help the diagnosis and treatment of PICCPs. Methods: Narrative review with multimodal imaging analysis. Results: Choriocapillaris non-perfusion can affect the end-choriocappilaries, at the benign end of the PICCP spectrum (MEWDS), to larger choriocapillaris vessels or precapillary vessels at the origin of more severe forms such as acute posterior multifocal placoid pigment epitheliopathy (APMPPE), idiopathic multifocal choroiditis (MFC) and Serpiginous Choroiditis (SC). Diagnosis is mostly based on multimodal imaging and especially on indocyanine green angiography (ICGA), fundus autofluorescence (FAF) and spectral-domain optical coherence tomography (SD-OCT)/OCT-angiography (OCT-A). ICGA shows the typical pattern of patchy lobular hypofluorescence reflecting hypo- or non-perfusion of the choriocapillaris that can also take the aspect of geographic areas in the more severe forms. Treatment depends on the severity of the disease and goes from observation in MEWDS and some mild cases of APMPPE, to oral corticosteroid and/or immunomodulator agents in the more severe conditions of APMPPE and MFC and SC cases. Close multimodal monitoring is crucial in order to introduce or adjust treatment. Conclusion: PICCPs are resulting from one common clinicopathological mechanism, inflammatory choriocapillaris hypo- or non-perfusion. ICGA findings are essential for the diagnosis and follow-up of PICCPs, but non-invasive methods such as FAF and SD-OCT/OCT-A also have their role especially in follow-up of the diseases. Treatment should be individualized according to the pathology and the evolution of lesions.

## 1. Introduction

Primary inflammatory choriocapillaropathies are a group of intraocular inflammatory diseases for which the site of inflammation is situated at the level of the choriocapillaris, producing choriocapillaris non-perfusion [1]. These conditions used to be included in the inappropriate and outdated misnomer of “white dot syndromes” including unrelated diseases and should be abandoned [2]. Depending on the extension and the sizes of capillary drop-outs, the spectrum of this group of diseases extends from benign and reversible, such as multiple evanescent white dot syndrome (MEWDS), to more severe involvement with non-reversible scars, such as acute multifocal placoid pigment epitheliopathy (APMPPE), idiopathic multifocal choroiditis (MFC) or serpiginous choroiditis (SC). Beside these well-defined phenotypes, overlapping and intermediary forms, as well as mixed forms having the aspect of one entity with the evolution of another, are completing the spectrum. For more than two decades, these conditions were classified with other unrelated syndromes in the potpourri group of “white dot syndromes” [2,3]. On the reverse, PICCPs have a common denominator, choriocapillaris non-perfusion. PICCPs have many other similarities: they generally affect young patients with a female predominance in most of them (Table 1), without any medical history although a viral prodrome can be mentioned before the initiation of ocular symptoms in a proportion of up to half of patients. Characteristics and epidemiological data of PICCPs are exposed on Table 1.

Patients often report photopsias and/or scotomas, and blurry vision. The symptoms are generally bilateral but asymmetric, except for MEWDS, which is mostly unilateral. However, some reports have described bilaterality [4]. Imaging findings are similar although the severity is different between the different conditions. The unifying feature is choriocapillaris inflammation, which can be demonstrated thanks to ICGA showing choriocapillaris hypo- or non-perfusion characterizing the group and explaining its pathophysiology [5,6]. Diagnosis can be challenging, as some of the diseases are overlapping and present with similar features [1,7]. Others are unclassifiable. Management is variable and depending on the type and severity of the disease. Our aim here was to apply pioneering pragmatism to clarify disease defining signs and diagnoses of these entities and redefine appropriate management and follow-up.

## 2. Diagnostic Imaging and Investigations

Nowadays, technology has provided the clinician with a large range of invasive and non-invasive imaging techniques allowing better visualization of the retina and the choroid [1,5,8]. After the crucial advent of indocyanine green angiography (ICGA) in the mid-1990 ties, more recently, new technologies such as fundus autofluorescence (FAF), spectral-domain optical coherence tomography (SD-OCT) and OCT-angiography (OCT-A) improved the appraisal of choroidal inflammatory diseases obtained by ICGA [8]. Before addressing the description of the diseases themselves, it is important to provide a summary of the imaging methods that contribute to their accurate diagnosis, which will be limited to an overview of the essentials.

### 2.1. Fundus Autofluorescence (FAF)

Blue-light fundus autofluorescence (BL-FAF) is a non-invasive retinal imaging method, which can demonstrate RPE and photoreceptor pathology even at an early stage of disease [9]. The autofluorescence signal derives from the normal lipofuscin accumulation in the RPE cytoplasm. In PICCPs, patchy and/or geographic hyperautofluorescence is the main finding caused by the loss of the photoreceptor outer segments. This reduces the photoreceptor screen, allowing us to better see the BL-FAF signal originating from the lipofuscin contained in RPE cells [1]. The non-invasive character of this exam makes it very useful in diagnosis, but more importantly in the follow-up of the inflammatory choriocapillaropathies, which in some cases, such as idiopathic multifocal choroiditis (MFC) and multiple evanescent dot syndrome (MEWDS), is a substitute to ICGA, in the follow-up of the patients.

### 2.2. Indocyanine Green Angiography (ICGA)

ICGA is the gold standard exam for visualizing the choroid in general and the choriocapillaris in particular, as it allows us to detect infrared choroidal fluorescence, bypassing the RPE barrier [5]. Furthermore, the ICG molecule is strongly bound (98%) to large size blood proteins constituting a large molecular complex with a molecular weight of 60,000 to 80,000 Daltons (d), which remains intravascularly except at the level of the choriocapillaris from which it egresses through the large fenestrations to fill the choroidal stroma. ICGA has helped to better understand the pathophysiology of choriocapillaritis diseases showing choriocapillaris non-perfusion of variable severity [10]. The angiographic translation of non-perfusion is represented by hypofluorescent areas of different extent and patterns. It should be avoided to use the term of hypo/hypercyanescence, a misnomer, as cyan means blue color, etymologically coming from the Greek word ‘κυανό’ (blue). Cyanesence, “becoming blue”, is an incorrect term, as the phenomenon is just ICGA fluorescence as it occurs in FA [11].

One advantage of ICGA consists in the fact that it can show very limited endcapillary patchy hypofluorescence/non-perfusion as is occurring in MEWDS. These are vessels with no or limited flow, therefore not accessible to OCT angiography (OCT-A) analysis. These patchy ICGA hypofluorescent lesions are better delineated in the late phases of angiography [5]. Detailed analysis and description of ICGA signs in choriocapillaritis have been published previously [1].

### 2.3. Spectral Domain-Optical Coherence Tomography (SD-OCT)

OCT is a non-invasive and easily repeatable method. As choriocapillaris non-perfusion and its consecutive ischemia directly affects the outer retina, especially the photoreceptors, OCT contributes to the diagnosis and follow up of PICCPs by showing the extent of secondary damage to the photoreceptor outer segments. The findings can be minimal, like interruption or swelling of the IS/OS line in some cases of MEWDS, to extended zones of atrophy in serpiginous choroiditis [1,5,8]. OCT is also helpful in detection of inflammatory choroidal neovascularization (ICNV) or macular oedema. 

### 2.4. OCT Angiography (OCT-A)

OCT-A is a non-invasive exam that has recently been added to our arsenal of exams [8]. It detects the presence or absence of flow in vessels in the retina and choroid. In the case of choriocapillaritis, it shows capillary drop out except in end-choriocapillary vessels, which cannot be imaged as there is an absence of significant flow. These vessels, thought to be at the origin of MEWDS, are not detected by OCT-A, and hence drop-out cannot be demonstrated. Therefore, this apparent absence of drop-out has been misinterpreted as choriocapillaris integrity, when it is only due to the fact that the methodology is inappropriate and cannot be applied to this situation. In the case of choriocapillaris drop-out of larger areas caused by involvement of larger vessels such as in APMPPE, MFC and SC, it is an important imaging tool for diagnosis, evaluation of the extent of lesions and follow up. For most instruments presently used in routine practice, the imaging is limited to the macula, but progressively new technologies appear with extended, more peripheral visualization. The patchy absence of choriocapillaris flow signal (choriocapillaris drop-out) shown by OCT-A in PICCPs does not contribute to a substantially better information than ICGA, except that it is non-invasive.

### 2.5. Fluorescein Angiography (FA)

Fluorescein angiography has a minor role in diagnosis of choriocapillaritis, as the choriocapillaris is visualized only in the first 60 s of the angiogram, where it shows choriocapillaris non-perfusion or perfusion delay. This sign allowed August Deutman [12] to give the appropriate explanation for APMPPE, choriocapillaris non-perfusion rather than an RPE pathology previously thought to be at the origin of the disease. In some severe cases of PICCPs, FA shows late hyperfluorescence due to pooling in the late angiographic frames that can be interpreted as a reactive dilatation and exudation from inner retinal vessels secondary to a profound ischemia of the external retina. 

### 2.6. Visual Field Testing and Microperimetry

Visual field testing shows functional impairment and corresponds to BL-FAF hyperautofluorescence and ICGA hypofluorescence and is useful for the functional follow-up. In subtle choriocapillaris, non-perfusion as seen in MEWDS of limited involvement visual field testing, may be normal and microperimetry is more sensitive to identify a decrease of retinal sensitivity. 

The most important imaging findings in PICCPs are presented in Table 2.

**Table 2:** Most important findings in multimodal imaging of PICCPs.

	MEWDS (Figure 1)	APMPPE (Figure 2)	MFC (Figure 3)	SC (Figure 4)
ICGA	Intermediate-late hypofluorescence	Early to late hypofluorescence	Early to late hypofluorescence (scars and new lesions)	Hypofluorescence with perilesional hyperfluorescence
BL-FAF	Hyperautofluorescence of acute lesions	Hyperautofluorescence in active lesions/hypofluorescence of severe acute and atrophic lesions	Hyperautofluorescence (active lesions) Hypoautofluorescence (atrophic lesions/scars)	**Active**: Hypoautofluorescence surrounded by hyperautofluorescent halo **Semi-Active**: linear hypoautofluorescence around hyperautofluorescent lesion **Inactive**: Hypoautofluorescence
SD-OCT	Loss of photoreceptor OS Interruption and/or damage to IS/OS line	Thickening and denaturation of outer retina (hyperreflective area occupying the outer retina)	Loss of photoreceptors OS/Interruption and/or damage to IS/OS line atrophic lesions/ICNV	Hyperreflective accumulations in the outer retina/ICNV
OCT-A	Absent or subtle signs of choriocapillaris drop-out	Patchy choriocapillaris drop-out	Patchy Choriocapillaris drop-out/ICNV	Geographic areas of choriocapillaris drop-out/ICNVs
FA	Subtle early to late hyperfluorescence	Early hypofluorescence/late hyperfluorescence (staining and pooling) in severe non-perfusion	Early hypofluorescence/late hyperfluorescence of scared-atrophic lesions No FA signs of new lesions	**Scars/atrophy**: early hypofluorescence and late hyperfluorescence (window defect) surrounded by late more hyperfluorescent rim, if lesions are progressing

BL-FAF = blue light fundus autofluorescence. ICGA = indocyanine green angiography. SD-OCT = spectral domain optical coherence tomography. OCT-A = OCT angiography FA = fluorescein angiography. ICNVs = inflammatory choroidal neovessels IS/OS = inner/outer segment line.

## 3. Clinicopathology of PICCPs

Until imaging investigation of the choroid became available, first thanks to ICGA, later followed by enhanced depth imaging OCT (EDI-OCT) and OCT-A, choroidal diseases such as PICCPs were ill-understood and difficult to classify. A first attempt at classification was undertaken in 1995 under the terminology of “white dot syndromes” (WDS) [13]. This classification, simply based on similar fundus lesions, was not only inappropriate, but at the origin of much confusion during the three decades that followed by grouping diseases not belonging together [2].

Once the choroid could be explored with substantial precision, it became clear that there were at least two main mechanisms and patterns of choroiditis, stromal infiltration by inflammatory foci on one side (stromal choroiditis) and inflammatory choriocapillaris non-perfusion (choriocapillaritis) on the other side. PICCPs fall under the latter mechanism. They are called primary because the trigger at the origin of the choriocapillaritis is unknown distinguishing them from forms where the trigger is identified, such as acute syphilitic posterior placoid choroiditis (ASPPC) or tuberculosis related serpiginous choroiditis, termed as secondary choriocapillaritis. The suspected trigger in PICCPs could be an unidentified virus or viruses, as a substantial number of patients present flu-like episodes before the ocular involvement. The patterns of PICCPs are most probably conditioned by the size of the vessels affected by the vaso-occlusive process and by its severity (Figure 5 and Figure 6).

Thus, a spectrum of diseases composes the group of PICCPs from discreet involvement when end-capillary vessels are occluded, such as in MEWDS, to more severe forms when larger capillary vessels or pre-capillary vessels are involved, such as in APMPPE, MFC and SC (Figure 7). In less severe forms such as MEWDS and mild forms of MFC and APMPPE, ischemia does not damage the more resistant RPE cell, but invariably affects the photoreceptor outer segments very sensitive to ischemia. In case of more severe ischemia, the process also affects the RPE cell and can lead to irreversible lesions and atrophic scars of photoreceptor outer segments, RPE and choriocapillaris and even full-thickness choroidal atrophy, as in severe forms of APMPPE and SC. The gold standard to detect non-perfusion is ICGA, showing patchy or geographic areas of hypofluorescence (non-perfusion). BL-FAF hyperautofluorescence co-localizes with these areas indicating loss of photoreceptor outer segments in these non-perfused areas which is morphologically shown on SD-OCT sections. When non-perfusion befalls larger choriocapillaris vessels and is sufficiently extended OCT-A shows choriocapillary drop out.

## 4. Treatment

Treatment strategy for PICCPs depends on the form of disease and its severity. On the benign end of the spectrum, MEWDS, can usually be followed and observed without treatment. Recommendation for APMPPE in textbooks is also forbearance of treatment. Indeed, observation without treatment can be practiced in case of mild disease. However, most of our APMPPE cases were pronounced and needed at least systemic corticosteroid therapy. In case of MFC and SC, dual or even triple immunosuppression is recommended. The diverse evolution of each single condition demands an individualized approach guided by close imaging follow-up and re-orientation of therapy if needed. For instance, if ICGA and/or BL-FAF follow-up of MEWDS shows a deterioration, therapeutical intervention can sometimes become necessary. In some cases, local sub-Tenon’s corticosteroid injections may be indicated if active disease is unilateral. Hereunder, we expose a quick overview of the agents and strategy used in our institution. 

### 4.1. Corticosteroid Therapy

Oral corticosteroid therapy still is the first line treatment in non-infectious uveitis, as it is able to rapidly put-out the inflammatory burst [14]. In PICCPs, oral corticotherapy is sufficient to reverse the pathology in some situations, while in more severe diseases such as SC, multiple immunosuppression is needed. Generally, treatment starts at 1 mg/kg with a relatively rapid tapering over 4–6 months. It is our trend not to give long-course steroids, but rapidly move on to non-steroidal immunosuppression. Use of oral corticosteroids should be limited because of their multiple side effects such as hyperglycemia, excitement, restlessness, osteoporosis, weight gain and the rare but deleterious side effect of psychotic evolution. Ocular side effects consist mainly of steroid-induced ocular hypertension and/or glaucoma, posterior subcapsular cataract development and central serous chorioretinopathy [15,16,17].

### 4.2. Immunomodulatory Agents

Immunomodulatory/immunosuppressive (IS) agents modify the immune response to obtain a decrease of an auto-immune response. Indications are (1) corticosteroid sparing in cases where corticosteroids cannot be tapered in reasonable time or when side effects require us to switch treatment, (2) in case of severe ocular inflammation not responding to corticosteroids alone or resistant to corticotherapy.

As for corticosteroids, it is important to rule out infectious causes of uveitis before the use of IS agents. In uveitis patients, COVID vaccination or any vaccination can exacerbate inflammation and, in reverse, patients under immunomodulatory therapy will respond sub-optimally to COVID or any other vaccination, and anti-COVID antibody levels should be monitored. Live vaccines should be proscribed. Hereafter, we present the agents we routinely use for PICCPs. The indications given hereunder reflect our personal routine use of immunosuppressives.

#### 4.2.1. Antimetabolites

Azathioprine is a purine analogue that interferes with DNA and RNA synthesis triggering, among other effects, apoptosis of T-cells. We usually start with a dosage of 2–2.5 mg/kg per day, but always less than 3 mg/kg. The advantage to start with a high dosage lies in the fact that it allows us to detect relatively more quickly whether the drug is effective or not when compared to the strategy of progressive increase of the dosage. It has to be stressed that azathioprine is a “slow starter” and needs 6–12 weeks to 4 months to be fully active. Side-effects mainly consist of upper gastrointestinal symptoms, and adverse effects include bone marrow suppression and hepatic toxicity [18,19]. Therefore, blood counts and hepatic laboratory tests should be performed every 2 months at the beginning of therapy and about every 4 months thereafter. Azathioprine can be at the origin of birth defects, but is considered moderately safe in pregnancy and lactation. The enzyme thiopurine S-methyltransferase (TPMT) is essential in transforming azathioprine into inactive metabolites. In approximately 1/300–400 patients, the enzyme is absent leading to severe toxicity. Therefore, at the start of treatment, there should be great caution to detect unusual intolerance to the drug. Some institutions test for the absence of TPMT before administering the drug [20]. Azathioprine, together with cyclosporine, tacrolimus and TNF-α inhibitors, are part of the World Health Organization’s list of essential medications, which is also justified because it is a cheap medication [21].

Mycophenolate mofetil (MMF, Cellcept^®^) inhibits the enzyme inosine-5′monophosphate dehydrogenase, stopping the purine biosynthetic pathways [22]. As a result, it has a strong cytostatic effect on lymphocytes (T + B), also decreasing antibody production of B-cells. The recommended dosage is 1–3 g per day. It is also a “slow-starter”, with full activity only several weeks after onset of therapy. It is better tolerated than azathioprine, even with maximal doses, with minimal side effects such as gastrointestinal discomfort, bone marrow depression and disturbed liver function tests [23].

Mycophenolic acid/sodium (Myfortic^®^) is an alternative form of mycophenolate. It comes as enteric coated tablets and is better absorbed than MMF, having also a better gastro-intestinal tolerance [24]. It is given at the dosage 720 mg twice a day. The mechanism of action of both forms of mycophenolate is identical and similar to azathioprine, being however more expensive [25]. Both forms should be stopped during pregnancy and lactation. Duration of treatment should be continued for at least two years.

#### 4.2.2. Calcineurin Inhibitors

Is a category of drugs which block T lymphocytes by suppressing the production of IL-2, a major enhancer for T-cell activation and recruitment. Cyclosporine (CsA) and tacrolimus (FK 506) are the two most commonly used agents of this category. The advantage of this class of immunosuppressive agents is that, unlike antimetabolites, clinical efficacy is reached quickly, within one (two) week and allows to taper corticosteroids in a timely fashion and to bridge the time lag of 2–4 months until antimetabolites reach their full efficacy [26].

##### Cyclosporine (CsA) (Sandimmun^®^)

CsA was first shown to be efficient in experimental autoimmune uveitis [27] and subsequently in uveitis in humans [28]. CsA initial dose is 2.5–5 mg/kg/day. We tend to start with the dosage of 4.5 mg/kg to be tapered after 4–6 weeks and discontinued after 5–8 months, as extreme caution must be applied to monitor renal function and arterial hypertension, the major adverse effects. Other side effects are dyslipidemia, hirsutism and gingival hyperplasia [29].

##### Tacrolimus (FK 506) (Prograf^®^)

Tacrolimus has the same mechanism of action as CsA, but has a better toxicity profile with at least similar if not better efficacy [26]. The initial dosage for Tacrolimus is 0.05–0.15 mg/kg per day. Side effects are similar to cyclosporine, but nephrotoxicity and hypertension are less common [30,31].

##### Strategy for Severe Choriocapillaritis

In case of MFC recurrence or SC, aggressive therapy must be applied. The protocol we are advocating is to start with triple immunosuppression as suggested by some authors [32], including systemic steroids (1 mg/kg) to obtain extremely rapid reduction of inflammation, tapered to 0 after 3–4 months, associated with cyclosporine (4.5 mg/kg), tapered to 0 over 5–8 months, and associated with mycophenolic acid (1440 mg/day) given for at least 2 years. Treatment must be monitored by ICGA and/or BL-FAF and readjusted in case of deleterious evolution with adjunction of biologic agents in case of unsatisfactory response. Before concluding that a treatment is inefficacious and opt for a change of immunosuppressive agent, it is recommended to measure serum trough levels of the immunosuppressant, although such measurements are difficult to interpret unless serum levels are obviously too low [33].

#### 4.2.3. Biologic Agents

There is limited and only anecdotal evidence of efficacy of biological immunomodulatory agents in choriocapillaritis. Infliximab was shown to be efficient in severe uveitis [34]. In PICCPs, mostly TNF-α blockers have been used. These are monoclonal antibodies producing death of cells that express and produce TNFα. The use of both infliximab (Remicade^®^) [35] and adalimumab (Humira^®^) [36,37,38,39] have been reported in MFC and SC in case reports and a small series of patients refractory to conventional immunosuppressive therapy. The agents were mostly used successfully with isolated exceptions. One study on MFC patients resistant to conventional therapy was especially demonstrative on the positive effect of adalimumab [37]. Active infections should be excluded in order to start treatment and it is crucial to rule-out previous contact with Mycobacterium Tuberculosis by performing an interferon gamma release assay (IGRA), as the use of TNF-α antagonists may be lethal in case of active tuberculosis. We both used adalimumab or infliximab with success in patients insufficiently responding to our triple IS conventional treatment protocol for MFC and SC. Dose, mechanism of action and side effects are presented in Table 3.

## 5. PICCPs Entities

### 5.1. MEWDS

MEWDS was first described by Jampol et al. in 1984 [41], and is the least severe form of choriocapillaris non-perfusion. The putative explanation is that only the distal part of the choriocapillaris net, characterized by almost absent or minimal flow, is occluded, without involving widespread areas of choriocapillaris nonperfusion (Figure 5). The fact that the choriocapillaris seems normal on OCT-A has been misinterpreted as absence of involvement of the choriocapillaris, whereas low or no-flow end-choriocapillaries can simply not be imaged by this technique based on the detection of flow. However, there are differences of severity within MEWDS cases and discreet choriocapillary drop-out or reduction of flow can be found by swept source OCT-A [42]. In contrast to OCT-A, ICGA shows small patchy areas of hypofluorescence, clearly identifying non-perfusion with consecutive ischemia. The loss and/or damage to the photoreceptor outer segments is due to ischemia and is not the result of primary involvement of the photoreceptors (photoreceptoritis), a hypothesis that has been put forward by some [43,44]. The body of evidence available and presented here is sufficient to classify MEWDS as a true primary choriocapillaritis [45]. The patients often present the triad of symptoms including photopsias, subjective scotomas and (not always) decreased visual acuity. Involvement is usually unilateral and predominantly affects middle-aged women. A flu-like viral episode precedes the ocular involvement in about half of patients.

Examination is characterized by absent or minimal anterior chamber inflammation. Fundus examination reveals at an early stage of disease faint yellowish-white dots in the posterior pole, around the optic disc and in the mid-periphery. However, some patients never present these lesions or they are no more present when the patients are consulting with some delay after the onset of symptoms. However, the most characteristic funduscopic finding is macular granularity, which can persist even when the rest of the fundus appears normal [46,47]. Diagnosis of MEWDS relies on the classical triad of (1) ICGA early patchy hypofluorescent areas, better delineated in the late angiographic frames, (2) BL-FAF patchy hyperautofluorescent areas co-localizing with ICGA lesions and (3) loss or damage to the photoreceptor outer segment IS/OS line on SD-OCT [47].

Among the investigations, ICGA is determining, showing patchy dispersed hypofluorescent areas in the early angiographic phase, much better defined in the late phase of angiography [1,47]. These hypofluorescent dots regress and become isofluorescent in the convalescent phase, indicating re-perfusion with remission of the disease. FA shows faint hyperfluorescent areas since the early angiographic phase corresponding to the ICGA hypofluorescent areas, a staining probably present in reaction to the ischemia of the outer retina. FAF shows characteristic patchy hyperautofluorescence, which corresponds to the areas of hypofluorescence on ICGA frames [1,47]. Hyperautofluorescence is detected because of the loss of the photoreceptor outer segment screen to physiological fluorescence of lipofuscin contained in the RPE cells. SD-OCT morphologically shows the damage or loss of the photoreceptor outer segments. OCT-A does not show the usual choriocapillary drop-out seen in more severe choriocapillaritis or only discrete areas of drop-out [40] (Figure 8b). It also shows CNVs, which are rarer than in other choriocapillaritis entities because of the small areas involved and the limited extension of ischemia [48].

**Scheme 1:**
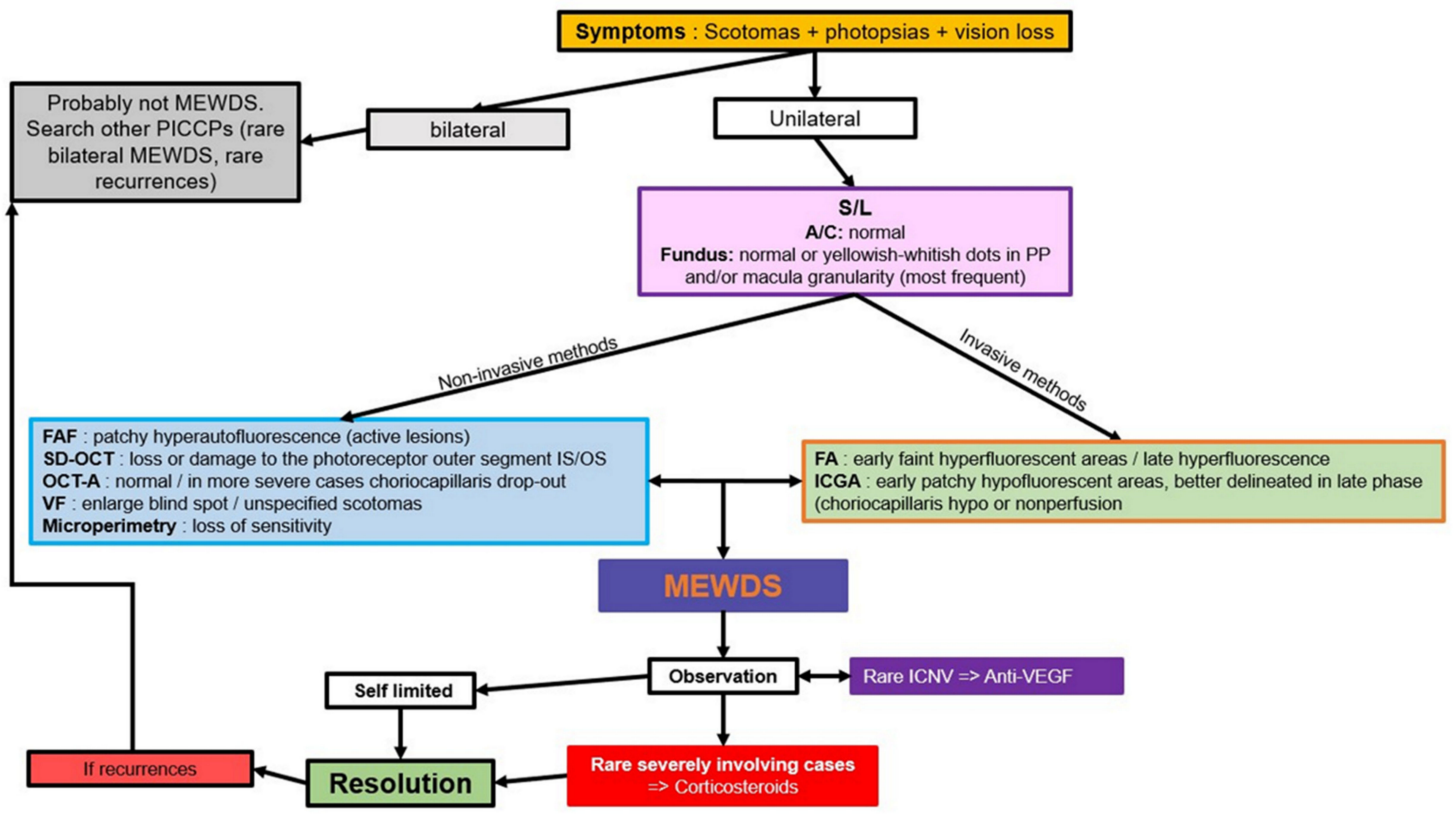
Appraisal of MEWDS: decision tree in clinical practice. MEWDS: Multiple evanescent dots syndrome. S/L: slit lamp. A/C: anterior chamber. FAF: autofluorescence. SD-OCT: spectral domain optical coherence tomography. OCT-A: OCT-angiography. VF: visual fields. FA: fluoresceine angiography. ICGA: indocyanine green angiography. ICNV: inflammatory choroidal neovascularization. Anti-VEGF: anti-vascular endothelial growth factor.

Complications of MEWDS include discrete to severe visual field disturbance on computerized visual field testing, which often shows an enlarged blind spot. A more subtle decrease of retinal sensitivity is shown by microperimetry. A particular form of MEWDS is acute idiopathic blind spot enlargement (AIBSE), which was shown to belong to the same pathology devoid of fundus findings [49,50]. As indicated, CNVs is a rarer complication in MEWDS compared to other diseases of the group such as MFC [51,52]. In most cases, MEWDS is spontaneously resolving and does not need to be treated. However, close follow-up is necessary with BL-FAF and SD-OCT, both non-invasive methods, in order to detect the rare cases with a progressing evolution, usually responding to systemic corticosteroid therapy. ICNVs need to be treated with anti-VEGF intraocular injections [53]. The notion of recurrent disease has to be considered cautiously, as MEWDS can be mistaken for the first episode of MFC, especially if scars are noted in “recurrent” episodes [7]. Appraisal of MEWDS is schematized on Scheme 1.

#### Illustrative MEWDS Case

A 27-year-old man complained of a sudden drop of vision with a subjective scotoma in his right eye. His best corrected visual acuity was 0.6 OD and 1.0 OS. Laser flare photometry was very slightly above normal amounting to 5.4 ph/ms OD, versus 3.5 ph/ms OS (normal values 3–5 ph/ms). Scarce cells were seen in the vitreous OD. The fundus showed faint whitish discolored areas barely visible. FA showed patchy hyperfluorescent areas in the posterior pole and mid-periphery from early to late frames, disc hyperfluorescence and faint peripheral retinal vasculitis. ICGA showed patchy hypofluorescence in the intermediate angiographic phase, much more clearly delineated in the late angiographic frame (Figure 8a) and BL-FAF showed hyperautofluorescence in the areas corresponding to ICGA hypofluorescence and loss of photoreceptor outer segments on SD-OCT (Figure 8a). The Octopus^®^ visual field was essentially normal, but microperimetry showed a decreased retinal sensitivity of 320/560 OD versus 492/560 OS (Figure 8b). OCT-A was almost normal with a few very small areas of capillary drop-out (Figure 8b). The retinal imaging fully normalized at 9 weeks.

### 5.2. APMPPE

APMPPE was first described by Gass in 1968, who attributed the lesion process to the RPE [54]. In 1972, however, Deutman rectified this hypothesis and understood that inflammatory choriocapillaris non-perfusion was at the origin of the disease [55]. He appropriately called the disease acute multifocal ischemic choriocapillaritis (AMIC) [56]. Deutman’s thesis of hypo- or non-perfusion was confirmed when ICGA became available, with an article published in 1995 [57]. As far as severity of lesions is concerned, APMPPE/AMIC seems to be in the middle range of the PICCP spectrum, as some cases can resolve without treatment while in others severe non-perfusion renders systemic corticosteroids and/or non-steroidal immunosuppression necessary [58,59,60]. Inflammatory occlusion affects larger choriocapillaris vessels, and, compared to MEWDS, the areas of non-perfusion are more widespread and confluent. It is a rare disease of young adults, possibly slightly predominant in men. As in other PICCPs, a flu-like viral episode may precede ocular involvement. The disease is bilateral and limited to one episode. If recurrences occur, the disease should be classified in the mixed intermediary forms [61]. Patients describe a blurry vision with subjective scotomas with or without photopsias. Symptoms can go from mild to very pronounced. Anterior segment involvement is not a rare thing and in some rare cases a significant non-granulomatous anterior uveitis with synechiae can occur (Figure 9a).

**Figure 2:**
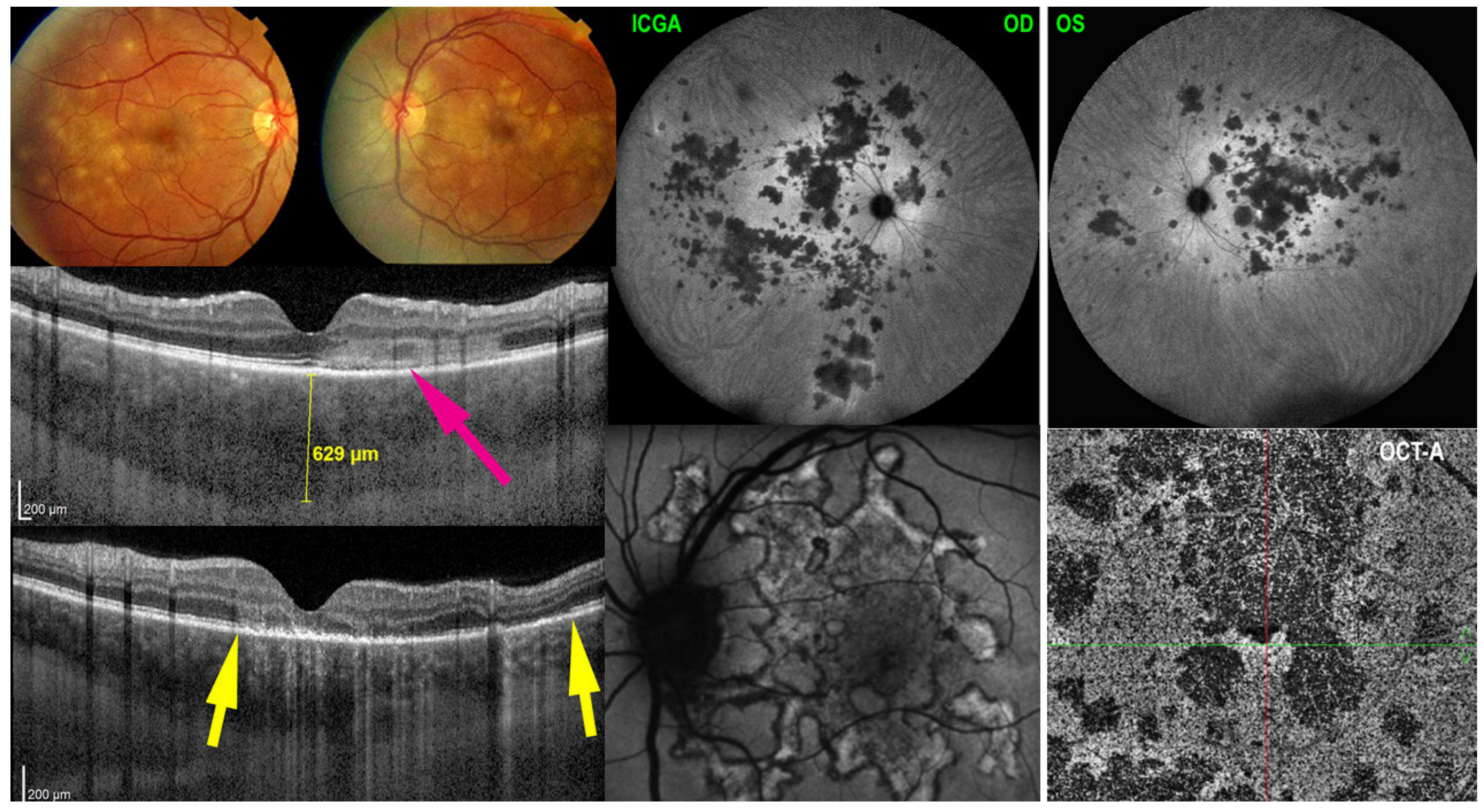
Multimodal imaging signs in APMPPE. Fundus (**top left**) shows numerous bilateral placoid discolored lesions, corresponding to ICGA hypofluorescent choriocapillary non-perfusion lesions (**top right** two frames). SD-OCT pictures show alteration and thickening of outer retina (ONL/photoreceptor IS/OS layer middle left picture, crimson arrow) and loss of photoreceptor IS/OS line (**bottom left** picture, between the two yellow arrows). BL-FAF shows hyperautofluorescence, indicating loss of photoreceptor outer segments as explained in paragraph 2.1 (**bottom middle** picture) OCT-A (**bottom right** picture) shows choriocapillary drop-out (dark areas).

Fundus examination reveals posterior pole areas of whitish “placoid” lesions in both eyes. Involvement can be asymmetrical, and there can be a delay from one eye to the other [54,61] (Figure 2). Diagnosis is helped by multimodal imaging and its findings derive from choriocapillary nonperfusion at the base of findings [1,5,8]. Infectious choroiditis has to be ruled out, especially acute syphilitic posterior placoid chorioretinitis (ASPPC), which can have the same presentation [62]. ICGA is the gold standard in the evaluation of APMPPE/AMIC and reveals large areas of scattered or confluent hypofluorescence in the early angiographic frames, present till the late frames [1,5,8]. FAF shows a mix of hyperautofluorescence, in areas with limited non-perfusion and limited ischemia, and of hypoautofluorescence in areas with severe non-perfusion having caused atrophy that include the RPE. In large lesions, hypoautofluorescence is central and hyperautofluorescence is located in the periphery of lesions where ischemia is less. SD-OCT findings vary according to the severity and the stage of the disease. In early stages, the outer retina ischemia that is provoked by choriocapillaris nonperfusion is presented by a “swelling” (hyperreflective zone) of outer retina going beyond the photoreceptor level (Figure 2). In later stages SD-OCT can reveal zones of absence of photoreceptors outer segment, impairment of IS/OS line or atrophy. Dilated inner retinal vessels reactive to outer retinal ischemia can be detected in early stages becoming less apparent in the convalescent stage [1,8,62]. Usually, the whole choroid is thickened, which is shown by enhanced depth imaging OCT (EDI-OCT) (Figure 2). OCT-A reveals choriocapillary drop-out, as the affected vessels are larger and give rise to larger confluent areas of non-perfusion. Areas of OCT-A drop-out correlate with the areas of ICGA hypofluorescence. FA contributes significantly to the understanding of disease mechanisms in APMPPE. It reveals early hypofluorescence, which corresponds to choriocapillary non-perfusion, which lead August Deutman [12] to propose choriocapillaris non-perfusion rather than RPE damage as the physiopathological explanation of APMPPE/AMIC. Another crucial FA finding is late hyperfluorescence due to pooling. The origin of the fluid is probably a response from the inner retinal vasculature to the outer retina ischemia and is probably associated with the finding of inner retinal vessels dilatation seen on SD-OCT [1,5,61] (Figure 9b).

**Scheme 2:**
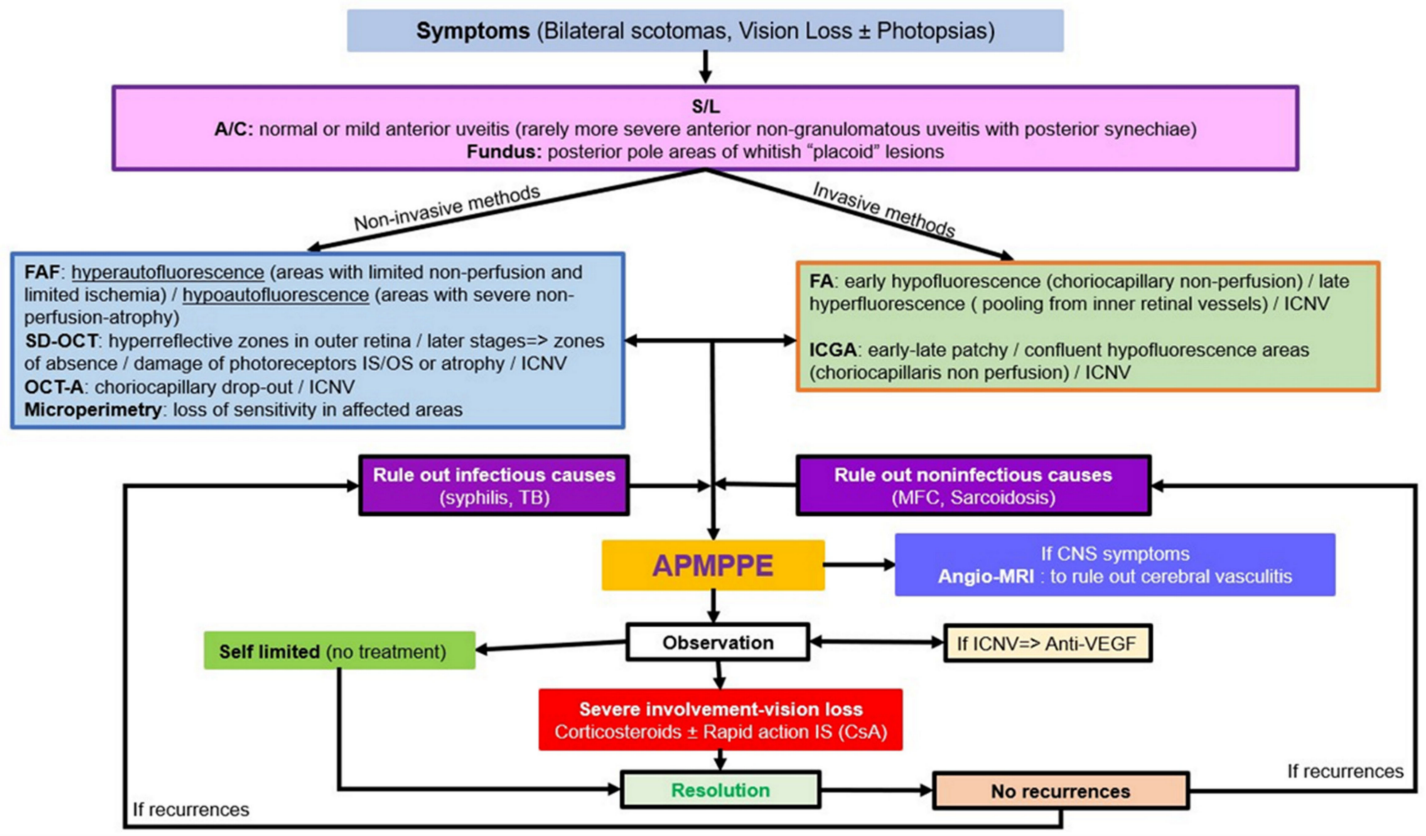
Appraisal of APMPPE/AMIC: decision tree in clinical practice. S/L: slit lamp. A/C: anterior chamber. FAF: autofluorescence. SD-OCT: spectral domain optical coherence tomography. OCT-A: OCT-angiography. VF: visual fields. FA: fluoresceine angiography. ICGA: indocyanine green angiography. TB: tuberculosis. MFC: multifocal. APMPPE: acute posterior multifocal posterior placoid epitheliopathy. CNS: central neural system. Anti-VEGF: anti-vascular endothelial growth factor. ICNV: inflammatory choroidal neovascularization. IS: immunosuppressors. CsA: cyclosporine.

Severity of involvement and, hence, prognosis for APMPPE cases is variable. In the literature, it is described as a self-limited condition. In our experience, however, many cases presented with extensive lesions and marked visual impairment and had to be treated with systemic corticosteroids, which seems to be sufficient as the insult is limited in time. We rarely had to resort to additional non-steroidal immunosuppression [58,59,60,63]. One complication or co-morbidity that the clinician should be aware of is cerebral vasculitis and in case of neurological symptoms, a cerebral angio-MRI should be performed [64,65]. Systemic vasculitis associated with APMPPE has also been reported [66]. Most numerous complications include chorioretinal scars and, as is always possible with scars, development of CNVs needing intravitreal anti-VEGF therapy. Appraisal of APMPPE/AMIC is schematized on Scheme 2.

#### Illustrative APMPPE/AMIC Case

A 28-year-old man was sent for an acute visual loss since 2 days predominant in his left eye. He reported a flu-like syndrome having started a few days earlier with a substantial elevation of serum c-reactive protein. He also described photopsias in both eyes. His BCVA was 1.0 OD and 0.6 OS. Laser flare photometry was 3.7 ph/ms OD and 4.4 ph/ms OS (normal values), and there was no sign of anterior segment inflammation. Fundus examination revealed multiple yellowish-white placoid lesions in the posterior pole and mid-periphery, being more widespread in the left eye.

SD-OCT of the left eye showed thickening of the photoreceptor outer segment layer (hyperreflective lesions) between the outer plexiform layer and the RPE associated with subretinal fluid (Figure 2 and Figure 10A). A remarkable sign seen on SD-OCT OS was the dilated vessels of the inner retina (Figure 10A, red arrows). Probably this vasodilatation can be explained by a response of retinal vessels to outer retina ischemia. SD-OCT OD also showed hyperreflective lesions of the outer retina (Figure 10B).

ICGA showed hypofluorescent patchy areas in the posterior pole mid-periphery, more coalescent around the fovea, which are present in early stages until late stages of the exam typical of APMPPE/AMIC. FA showed a mixture of hypofluorescence and hyperfluorescence depending on the severity of non-perfusion areas. FAF, similarly, showed hyperautofluorescence along the superior arcade and zones of hypoautofluorescence around the fovea (Figure 11). OCT-A showed characteristic choriocapillaris drop-out in both eyes (Figure 12).

Blood tests were done to rule out any infectious cause such as syphilis or tuberculosis. Imaging features and the negative blood markers confirmed the diagnosis of APMPPE/AMIC. Treatment was started with oral prednisone 1 mg/kg/day and cyclosporine 4 mg/kg as lesions were occupying the posterior pole and especially the macula of the left eye (Figure 11C). OCT-A showed a favorable evolution with reperfusion of many areas but remaining areas of choriocapillaris drop-out (Figure 12).

### 5.3. Idiopathic Multifocal Choroiditis (MFC/PIC)

**Figure 3:**
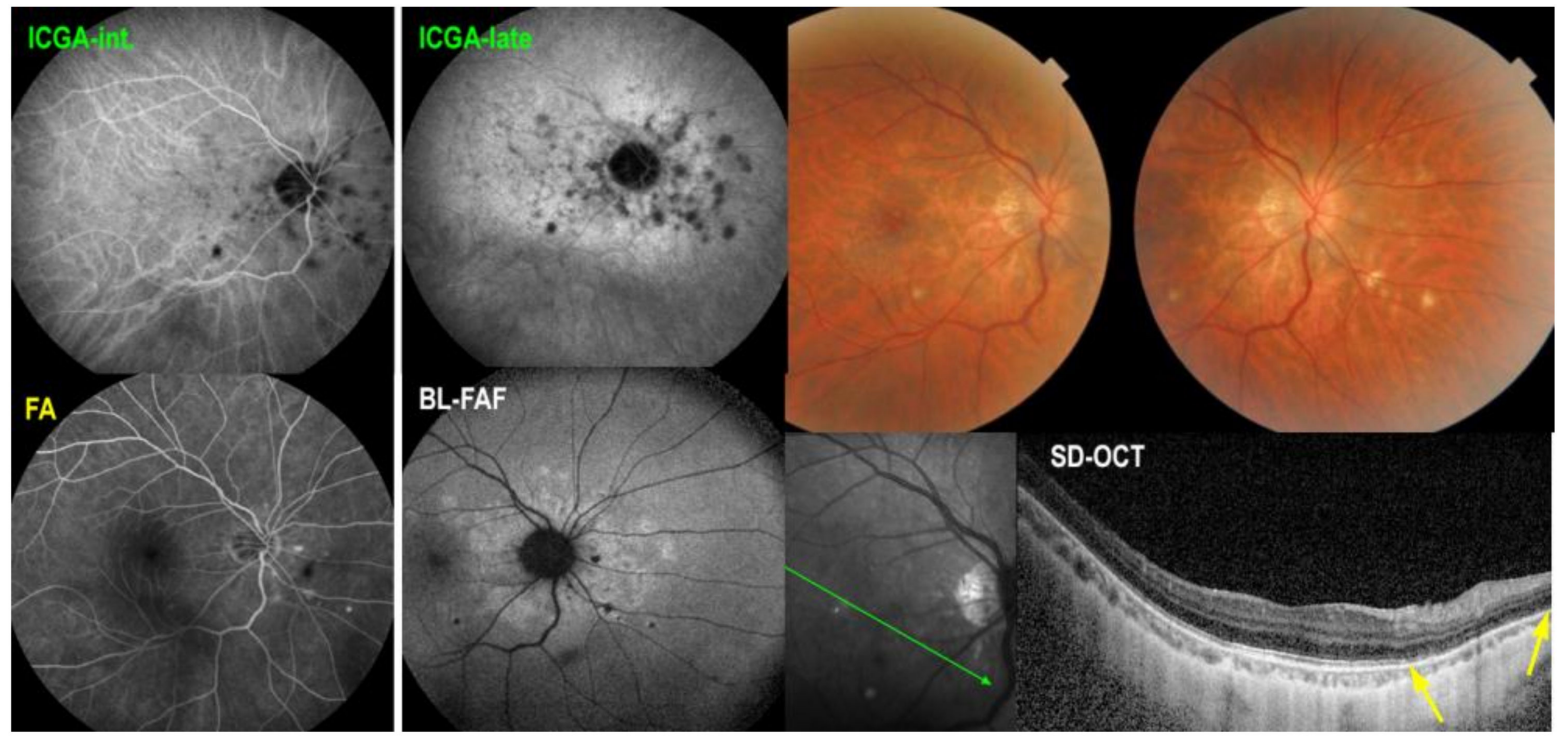
Multimodal imaging signs in MFC. The ICGA (**top** two left frames) show numerous hypofluorescent dots in the intermediate angiographic phase, (**top left**) much better visualized in the late phase frame (ICGA-late). Some hypofluorescent lesions correspond also to the cicatricial lesions visible as hyperfluorescent punctiform spots on fluorescein angiography (**bottom left**). The BL-FAF picture (**bottom middle**) shows hyperautofluorescent areas corresponding to active lesions characterized by loss of photoreceptor outer segments shown on SD-OCT (**bottom right**, between two yellow arrows).

Idiopathic multifocal choroiditis (MFC) is a terminology that today merges several entities that were considered separately in the past. Since a consensus nomenclature article was published in 2013 [67], multifocal choroiditis and panuveitis, punctate inner choroiditis (PIC) and pseudo-POHS (presumed ocular histoplasmosis syndrome) and others are considered as one and the same disease termed idiopathic multifocal choroiditis. In the past the term of MFC and panuveitis was used [68]. However, in our experience, panuveitis was present in none of our cases and cannot be considered as a disease defining feature. This was also reported in a large series by others [69]. The form of choriocapillaris non-perfusion in MFC resembles MEWDS with similar multimodal imaging features. However, the lesion process is more extensive, non-resolving and often bilateral, usually sequential in one and the other eye with recurrences [70]. Lesions often leave scars. Due to the numerous scars and repeated ischemic attacks, ICNVs develop in a relatively higher proportion (up to 30%) compared to the other PICCPs [71]. MFC affects in majority young to middle-aged myopic women. Patients present with photopsias and decreased vision if the lesions are foveal or parafoveal and visual field changes. Fundus examination allows us to distinguish two categories of lesions: (1) punched out small chorioretinal scars in the posterior pole and mid-periphery and sometimes also in the periphery [72] from previous unnoticed episodes and (2) larger faint yellowish-white lesions localized in the posterior pole and mid-periphery resulting from a novel active acute MFC attack [1,5,61,70] (Figure 3).

Diagnosis is much easier, disease defining features more clearly established, and their monitoring more precise since multimodal imaging is available. During an active phase of the disease, ICGA reveals two types of lesions, (1) small areas remaining hypofluorescent during the whole angiographic sequence (hyperfluorescent on FA) imaging chorioretinal scars of previous episodes and (2) larger hypofluorescent areas from early to late angiographic frames, not visible on FA and delineating new areas of choriocapillaris non-perfusion [1,5,61,68] (Figure 3). FAF is very sensitive in MFC and presents with areas of hyperautofluorescence (active lesions) and hypoautofluorescent spots which correspond to the atrophic scars of previous episodes. FAF is a valuable follow-up modality, as it is a non-invasive and, in some instances, more sensitive than ICGA [1,8,61,68] (Figure 3). FA shows hyperfluorescent spots seen throughout the angiographic sequence which correspond to the chorioretinal scars producing a window defect. However, FA does not show the new active occult lesions only detected by ICGA. SD-OCT findings are similar to other PICCPs with impairment of IS/OS line and damage and/or loss of the photoreceptor outer segments and zones of chorioretinal atrophy. OCT is also helpful in the detection of ICNV, much more clearly shown on OCT-A. OCT-A findings are characterized by choriocapillary drop-outs of limited size as well as detection of ICNV if present. VF shows scotomas corresponding to the new acutely involved areas which regress under treatment in parallel with resolution of ICGA hypofluorescent areas [70]. As in all PICCPs, VF can also show enlargement of the blind spot [73].

**Scheme 3:**
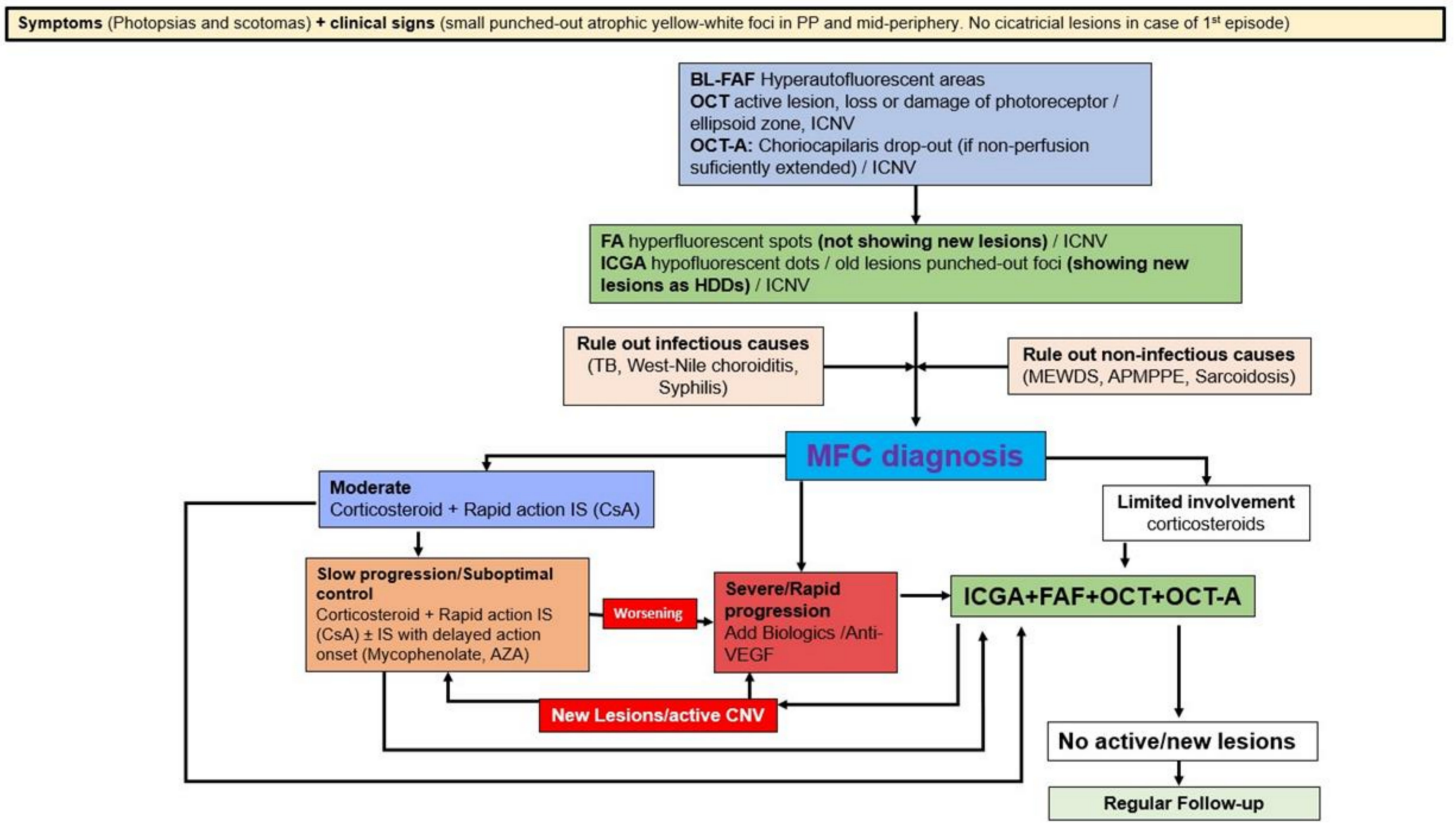
Appraisal of MFC: decision tree in clinical practice. PP: posterior pole. OCT: optical coherence tomography. iCNV: inflammatory choroidal neovascularization. BL-FAF: blue light autofluorescence. OCT-A: OCT-angiography. FA: fluorescence angiography. ICGA: indocyanine green angiography. HDDs: hypofluorescent dark dots. TB: tuberculosis. MEWDS: multiple evanescent white dots syndrome. APMPPE: acute posterior multifocal placoid pigment epitheliopathy. MFC: idiopathic multifocal choroiditis. IS: immunosuppressors. CsA: Cyclosporine. AZA: Azathioprine.

MFC is not resolving spontaneously and can have a deleterious evolution with a higher propensity to develop ICNVs if left untreated. Moreover, recurrences have to be feared in the absence of treatment [74]. Aggressive immunosuppressive therapy is recommended [75]. We apply our empirical triple immunosuppressive strategy including corticosteroids for immediate inflammation-suppressive effect, tapered within 4–5 months to avoid the numerous steroid-induced side-effects. This is coupled with a rapidly acting immunosuppressant of the calcineurin-inhibitor type, tapered within 6–8 months together with a “slow-starter” but well-tolerated anti-metabolite such as mycophenolate, or azathioprine if cost problems intervene. TNF-α inhibitors have been shown to be an alternative if conventional immunosuppression is insufficient and/or ineffective [37]. In case of ICNVs, intravitreal injections of anti-VEGF agents have to be given additionally [76]. ICGA and BL-FAF are essential to monitor the evolution of the disease. The recovery of structures is morphologically monitored by OCT. Detection and follow-up of ICNVs is best achieved by angiography and by the non-invasive OCT-A modality. Appraisal of MFC is schematized on Scheme 3.

#### Illustrative MFC Case

A 40-year-old myopic woman presented with a paracentral scotoma in her left eye (OS) since one week. Ophthalmic history included a vitrectomy for rhegmatogenous retinal detachment in her right eye (OD) and cataract surgery in both eyes. Myopic spheric equivalent refractive values prior to cataract operation were 11.75 D in the right eye and 10 D in the left eye. At presentation, BCVA was 0.9 OD and 0.8 OS. No signs of inflammation were detected in the anterior chamber, but laser flare photometry (LFP) values were slightly elevated to 9.5 ph/ms OD and 13.2 ph/ms OS. There were no cells visible in the vitreous. Fundus examination of OS showed pale discolored dots around the optic disc and along the superior temporal arcade. These lesions corresponded to faintly hyperfluorescent areas on FA. On ICGA, there was extensive unilateral peripapillary hypofluorescence extending inferiorly (Figure 13a top). Additionally, there were patchy ICGA hypofluorescent dots along the superior temporal arcade. FAF showed unilateral patchy hyperautofluorescent areas corresponding to the ICGA hypofluorescent areas (Figure 13a bottom). SD-OCT showed areas of photoreceptors IS/OS damage, all characteristic findings of MEWDS. One year later, however, the right eye developed a similar episode when the left eye did not present any activity (Figure 14). Upon examination of the previously affected left eye, there were several small chorioretinal scars visible. The bilaterality of the findings and the presence of small scars were in favor of MFC. The diagnosis was rectified to MFC of which the original “MEWDS” episode in the left eye was in fact the inaugural MFC episode.

Treatment initially was local, with sub-Tenon’s corticosteroid injections (triamcinolone acetonide, 40 mg) around the affected eye as the patient refused systemic treatment.

Several recurrences occurred, especially in the left eye (Figure 14) as periocular treatment had a limited duration.

The patient finally accepted systemic treatment. Oral corticosteroids starting at 1 mg/kg/d were given in parallel with CsA and mycophenolic acid and were tapered to 0 within 4 months. After 11 years of follow up, BCVA remained at 0.9 OD and 1.0 OS and with disease inactivity for more than 3 years under systemic treatment (Figure 15).

### 5.4. Serpiginous Choroiditis (SC)

**Figure 4:**
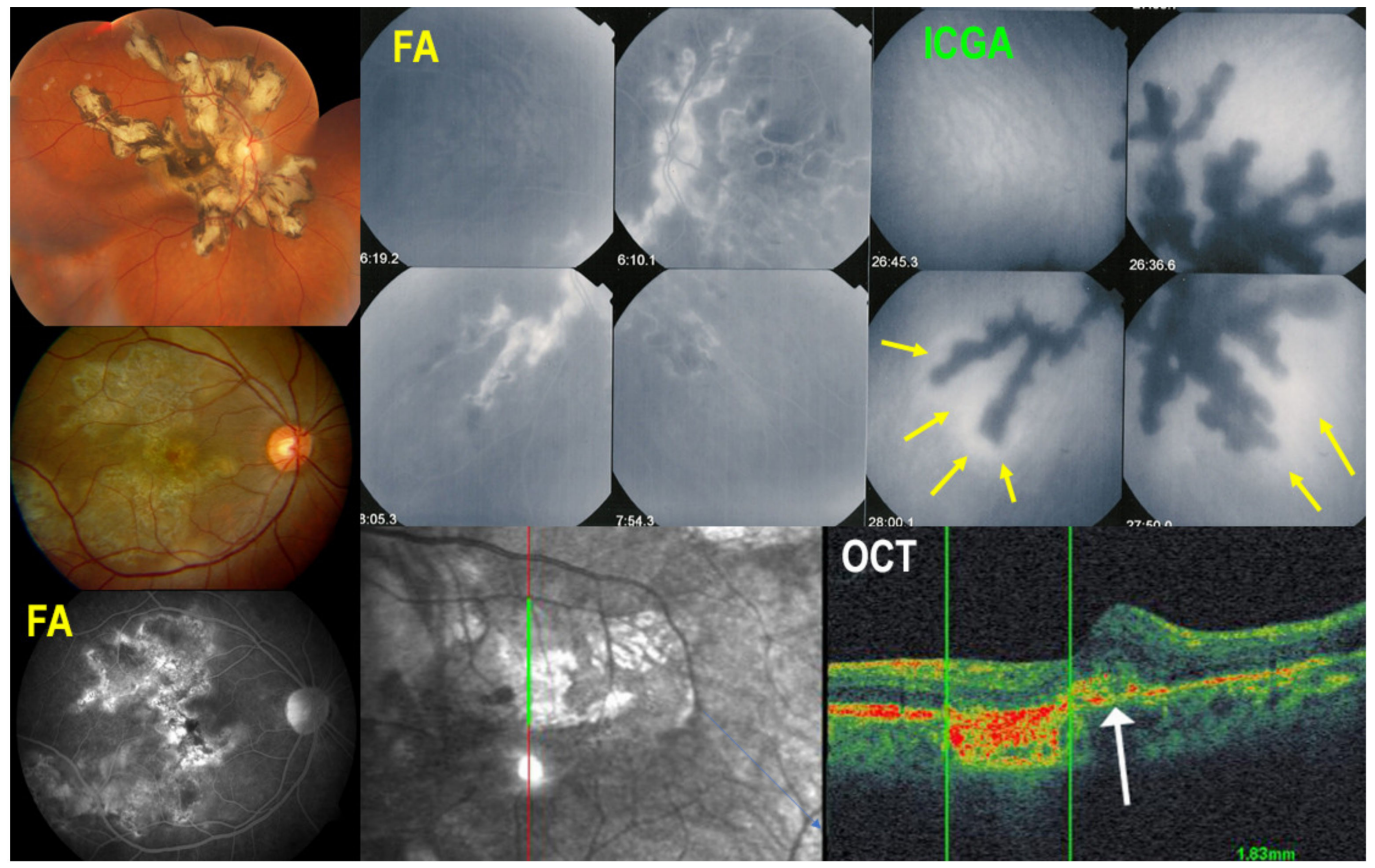
Multimodal imaging signs in SC. Typical fundus aspect of SC (**top left**); extensive central atrophy in a different case (**middle left**) with FA hyperfluorescent window effect (**bottom left**). Comparison of FA (**top middle**) and ICGA (**top right**) findings; ICGA hypofluorescence is much more widespread than FA hyperfluorescence, as it represents both atrophy and choriocapillaris non-perfusion of new lesions. Perilesional ICGA hyperfluorescent halo (yellow arrows) indicates progression of disease. OCT (**bottom right**) shows that at the border of atrophy, there is retinal oedema and damage to the outer retina (white arrow).

**Figure 5:**
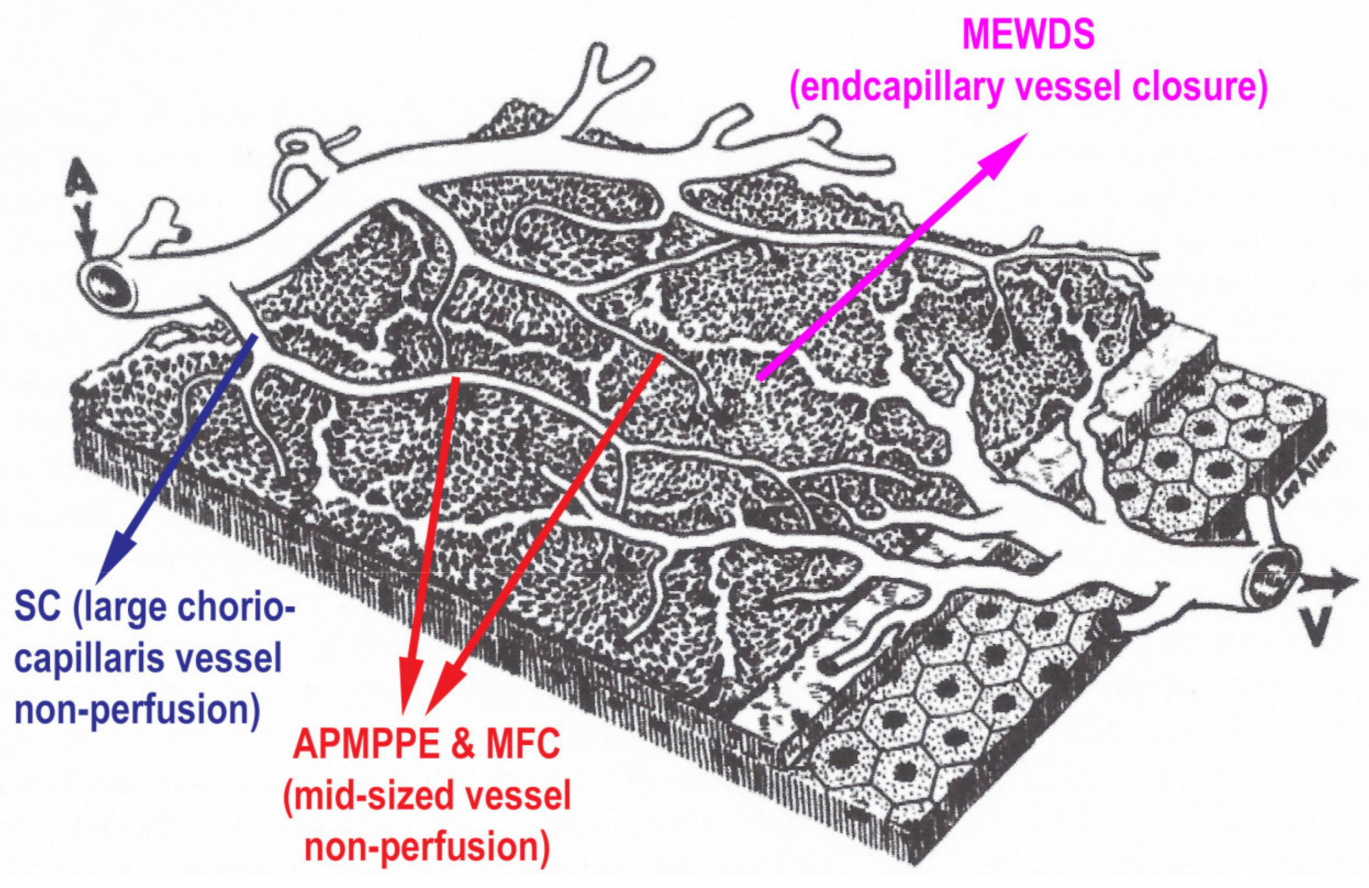
Clinicopathology of PICCPs according to vessel location of non-perfusion. Schematic drawing of the different levels of choriocapillaris vessels, the occlusions of which determine the type of choriocapillaritis. (Reproduced from Hayreh SS, Br J Ophthal 1973; 59:631, by permission of the BJO).

**Figure 6:**
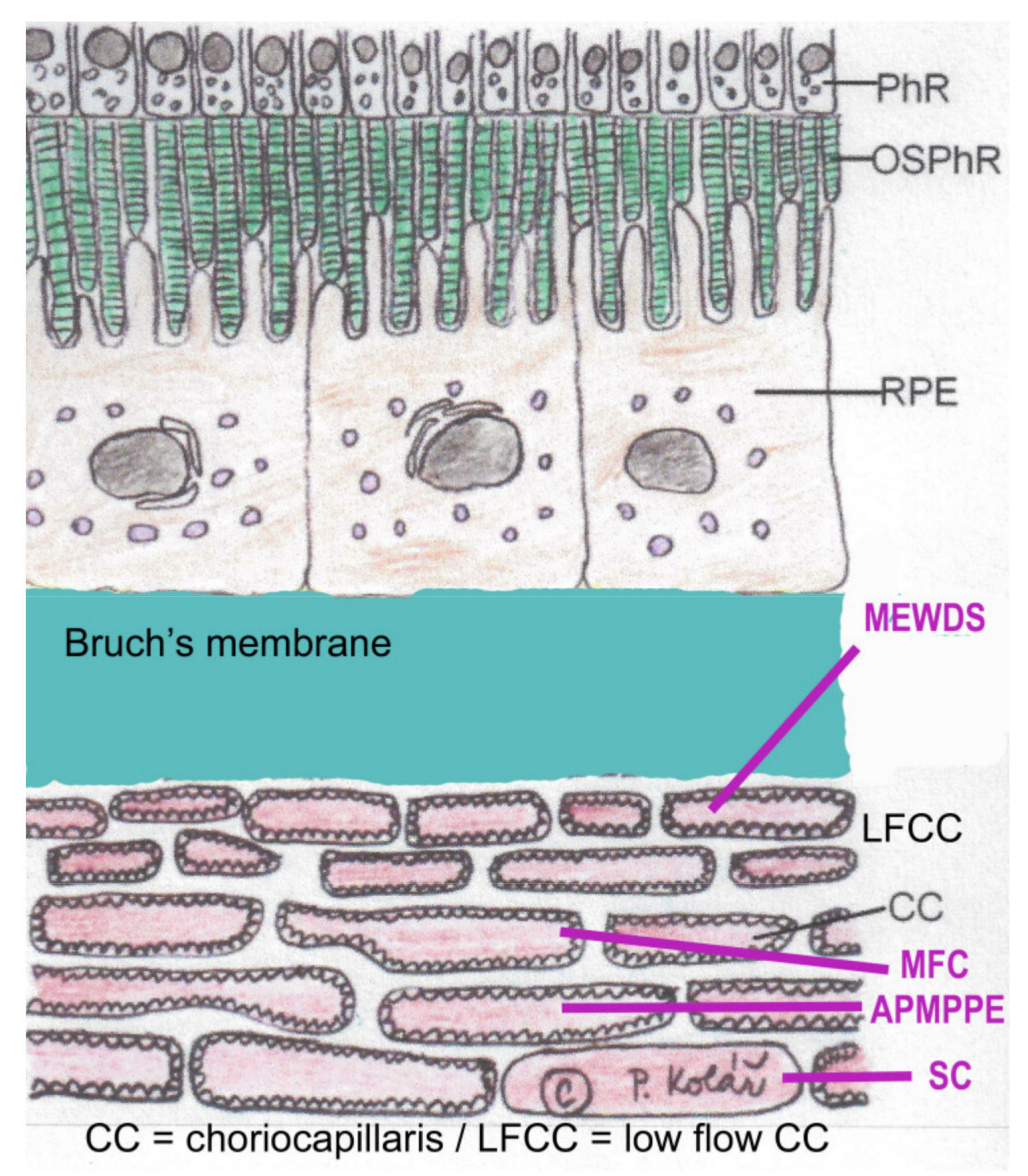
Clinicopathology of PICCPs according to size of non-perfused vessel. Schematic representation of the choriocapillaris with inner low-flow choriocapillaris (LFCC), the occlusion of which is at the origin of MEWDS. Occlusion of larger choriocapillaris vessels (CC) are at the origin of more severe conditions such as APMPPE, MFC or SC. (Reprinted by permission and modified from Petr Kolar, DOI:10.5772/53762) PhR = photoreceptors; OSPhR = outer segments of photoreceptors; RPE = retinal pigment epithelium.

**Figure 7:**
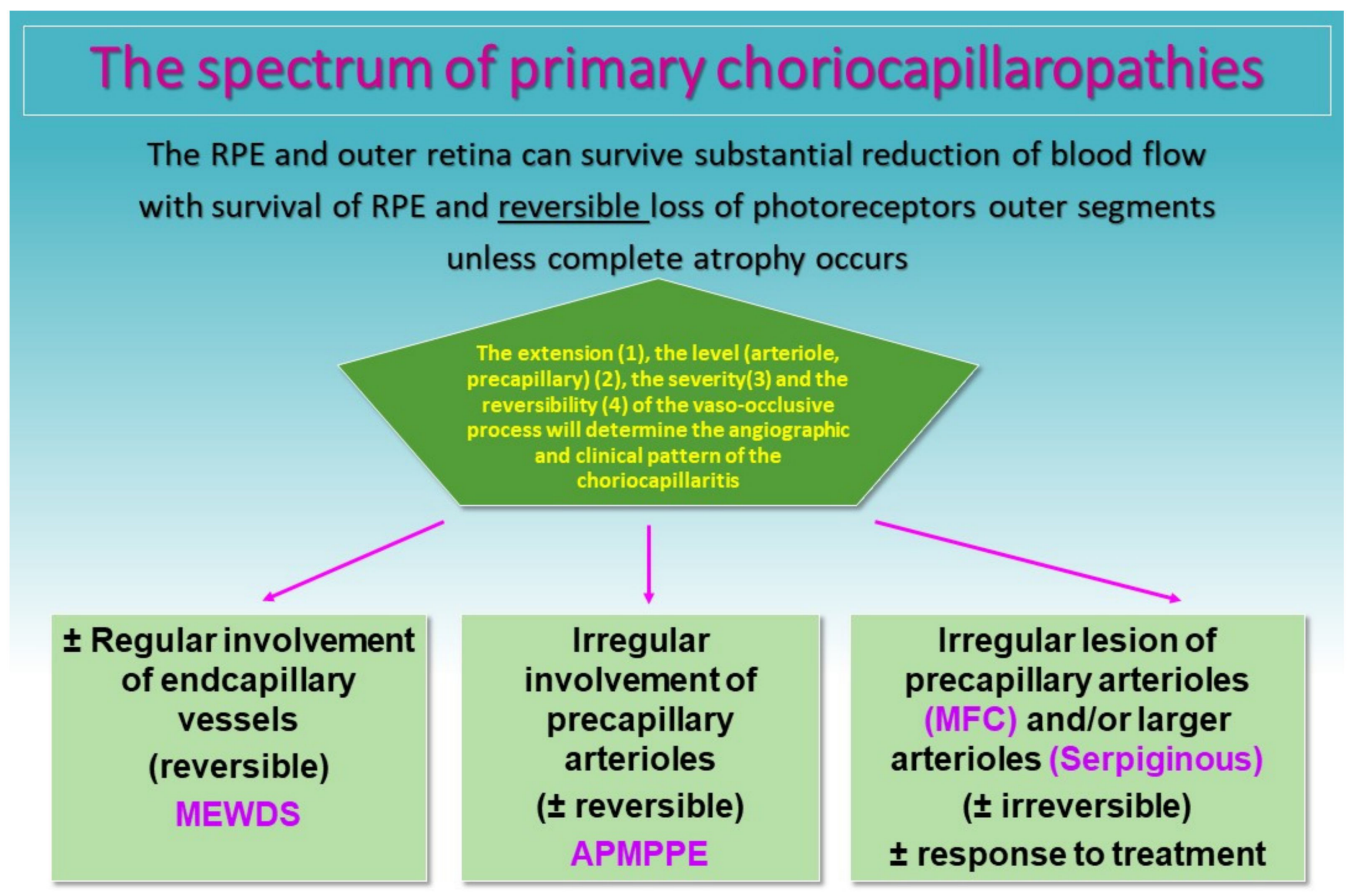
Clinicopathology of PICCPs according to the suspected type and extension of vessel non-perfusion. Non-perfusion of low-flow endcapillary vessels cause the benign and reversible disease MEWDS. If larger choriocapillaris vessels or precapillary vessels are occluded the more severe diseases develop including APMPPE, MFC and SC.

**Figure 8:**
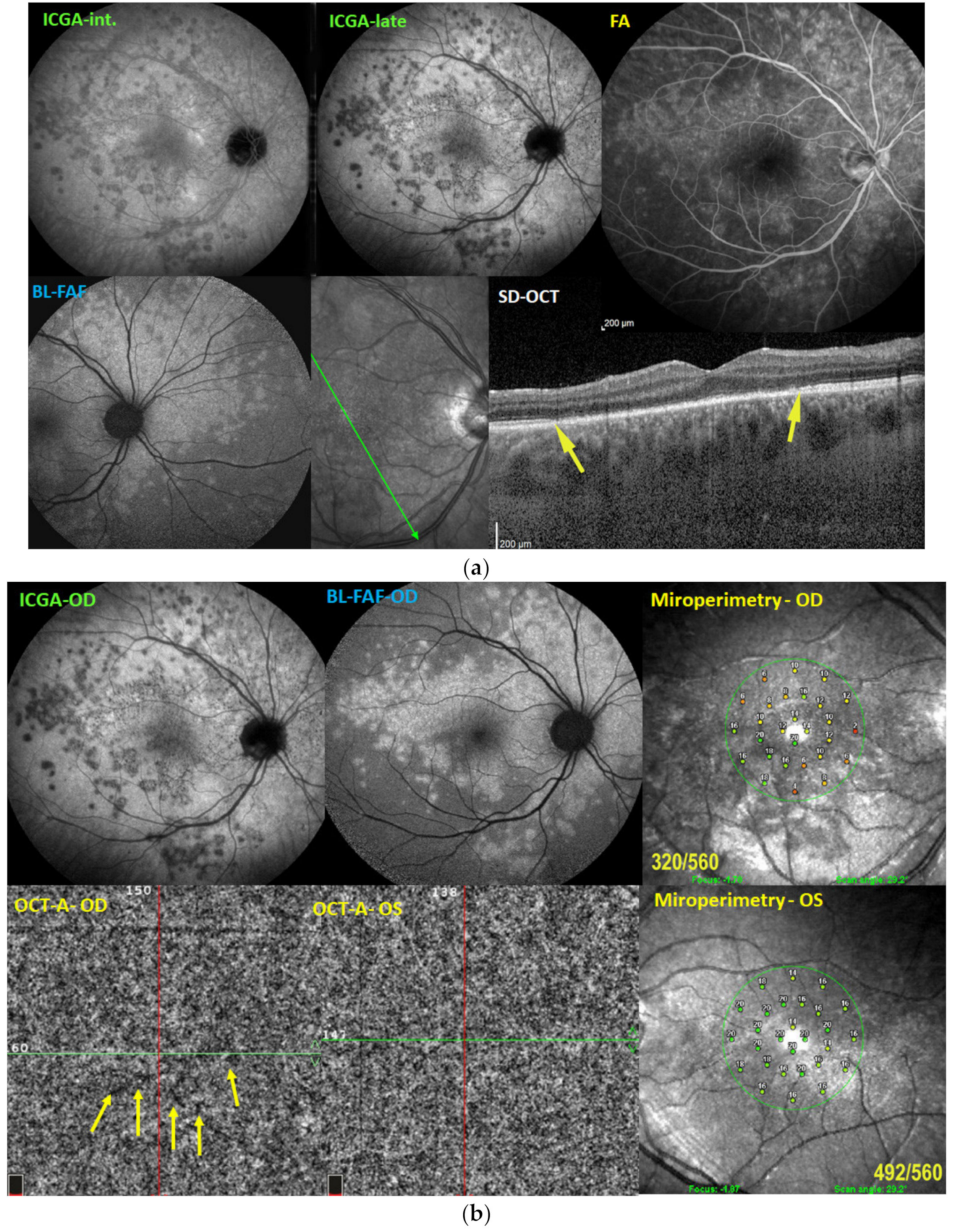
(**a**) Illustrative case of MEWDS, multimodal imaging. Scattered areas of hypofluorescent dots seen on intermediate angiographic phase of ICGA (ICGA-int, **top left**), much better identified on the late phase frame (**top middle**, ICGA-late). FA (**top right**) shows faint hyperfluorescence in the same areas. BL-FAF shows hyperautofluorescent area corresponding to ICGA hypofluorescence due to loss of photoreceptor outer segments and better visualization of the normal RPE lipofuscin autofluorescence. Loss of photoreceptor outer segments is shown on the SD-OCT section between the yellow arrows (**bottom right**). (**b**) Illustrative case of MEWDS: OCT-A and microperimetry. Extensive areas of end-capillary non-perfusion on ICGA (**top left**) corresponding to hyperautofluorescence (**top-middle**) due to loss of photoreceptor outer segments as explained in paragraph 2.2. OCT-A of the MEWDS eye (OD) shows tiny areas of choriocapillary drop-outs. Although the Octopus^®^ visual field was normal, microperimetry of the MEWDS eye showed a decreased retinal sensitivity (**top right**) compared to the fellow normal eye (**bottom right**).

**Figure 9:**
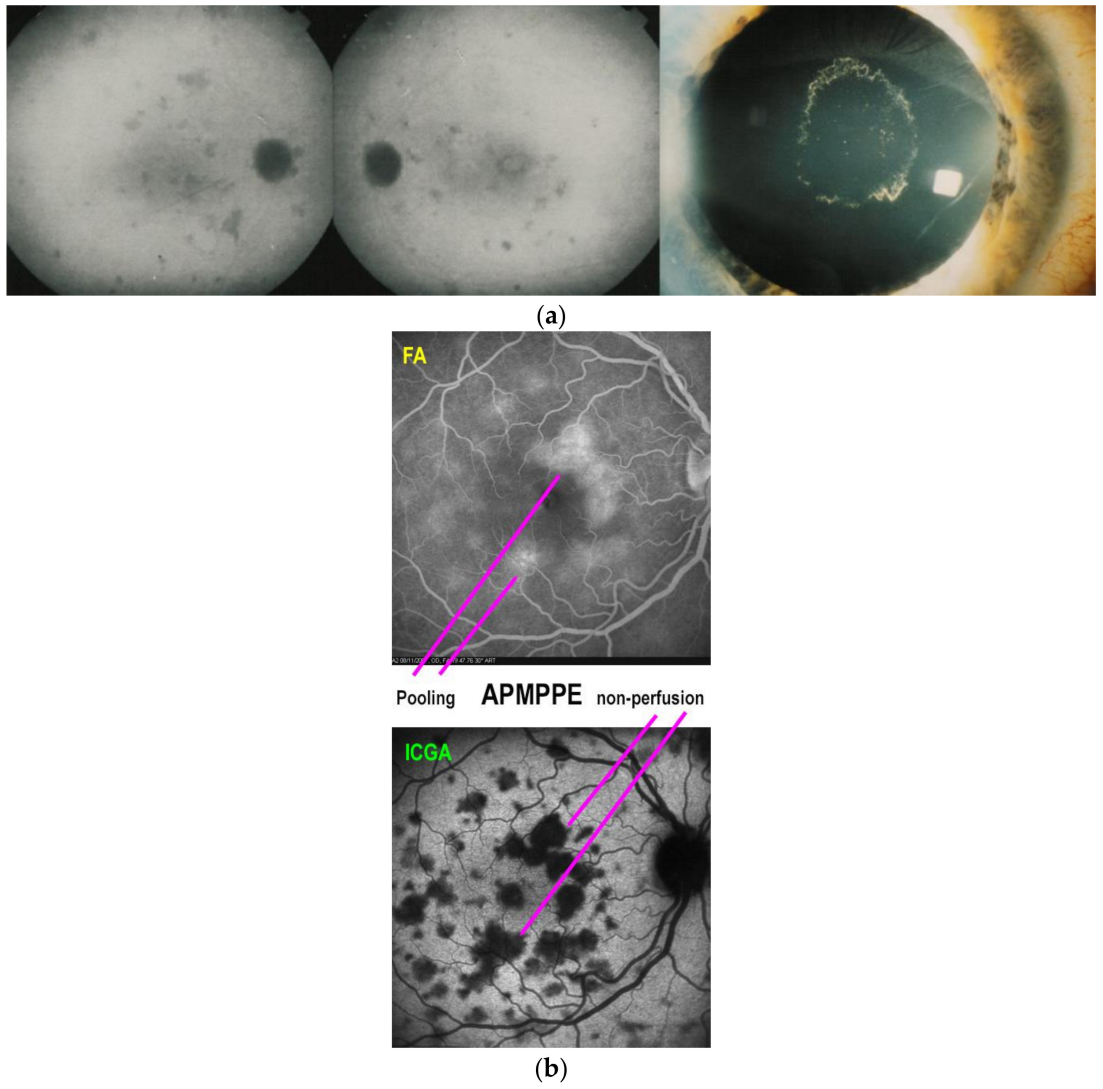
(**a**) Case of APMPPE/AMIC with associated anterior uveitis. This patient presented with a bilateral non-granulomatous anterior uveitis with synechiae (**right** picture), a rare occurrence in APMPPE. The fundus showed bilateral placoid yellow lesions in the posterior pole (not shown) and discreet areas of ICGA hypofluorescence (**left** and **middle** pictures) that resolved without scars (not shown) following a short course of 40 mg of prednisone, tapered over 5 weeks. (**b**) Late FA pooling in a severe case of APMPPE/AMIC. In severe cases of APMPPE/AMIC, FA shows areas of abundant retinal pooling in the late angiographic phase (**top** picture). Traditionally this phenomenon is explained by the very hypothetical alleged change of polarity of the RPE and fluid movement from the choroid to the retina. However, the choriocapillaris areas under the retinal pooling are non-perfused (**bottom** picture). Therefore, a more probable origin of the fluid is exudation from retinal vessels in response to severe outer retinal ischemia.

**Figure 10:**
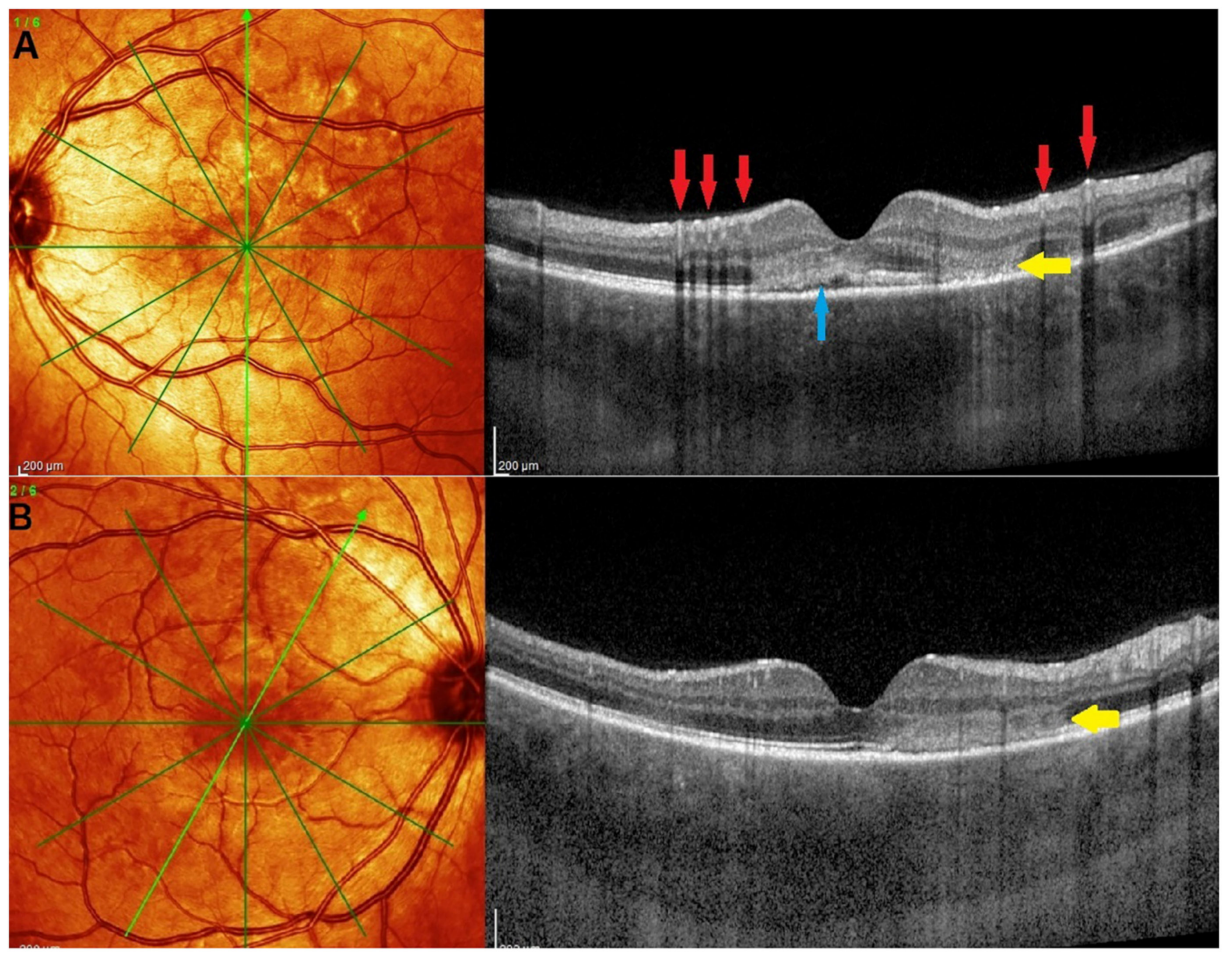
OCT of a patient with APMPPE. (**A**) OCT of left eye: Ischemia of outer retina is presented as an hyperreflective lesion (yellow arrow). A zone of subretinal fluid is also detected (blue arrow). Dilatation of inner retinal vessels leaving a shadow (red arrows). (**B**) OCT of the right eye also showed an hyperreflective zone in the outer retina (yellow arrow).

**Figure 11:**
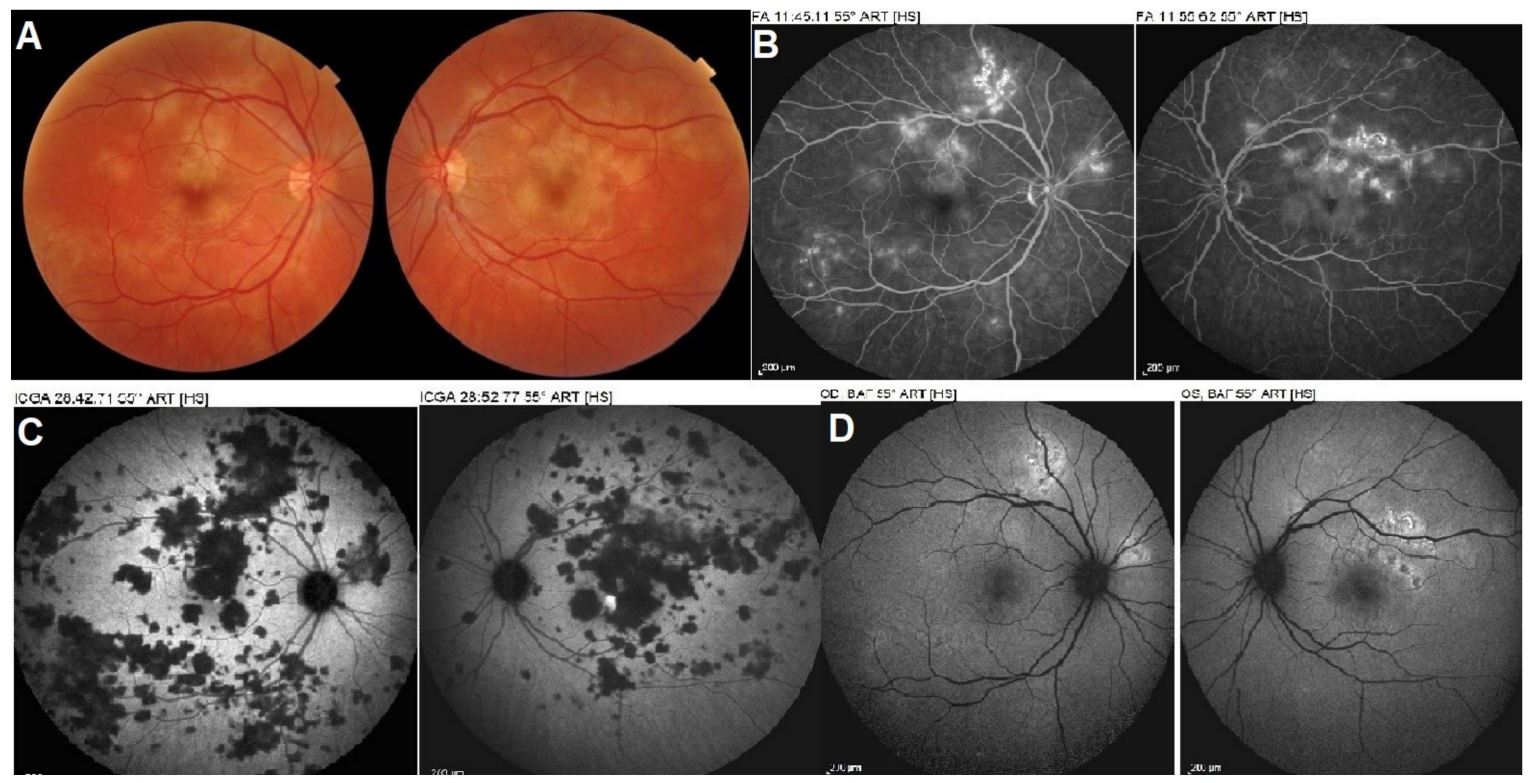
Multimodal imaging of APMPPE/AMIC. (**A**) Bilateral fundus pictures revealed whitish placoid lesions in the posterior pole and mid-periphery. (**B**) Same day late phase FA frames showed hyperfluorescence probably due to inner retinal vessel exudation. (**C**) ICGA late frames showed characteristic hypofluorescent patches, more widespread than the pathologic lesions seen on FA and BL-FAF (**D**).

**Figure 12:**
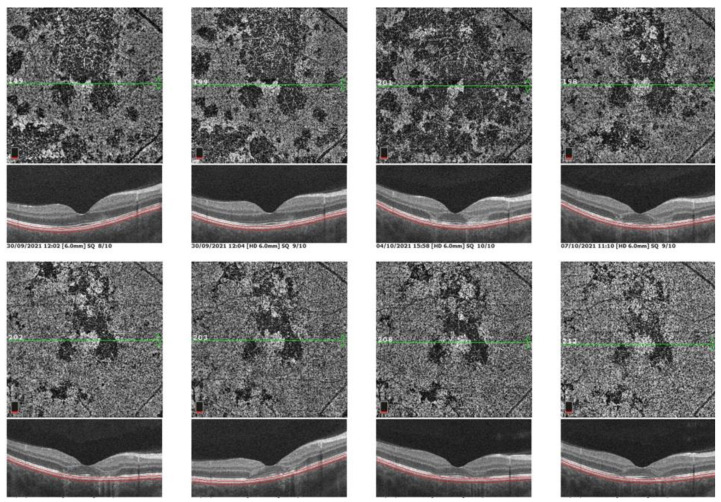
Sequence of OCT-A frames from presentation to convalescent stage. OCT-A 6 × 6 en face scan of the posterior pole OD choriocapillaris showing progressive resolution from the first scan (**top left**) to last scan (**bottom right**). Dark areas of non-perfusion were partly re-perfused.

**Figure 13:**
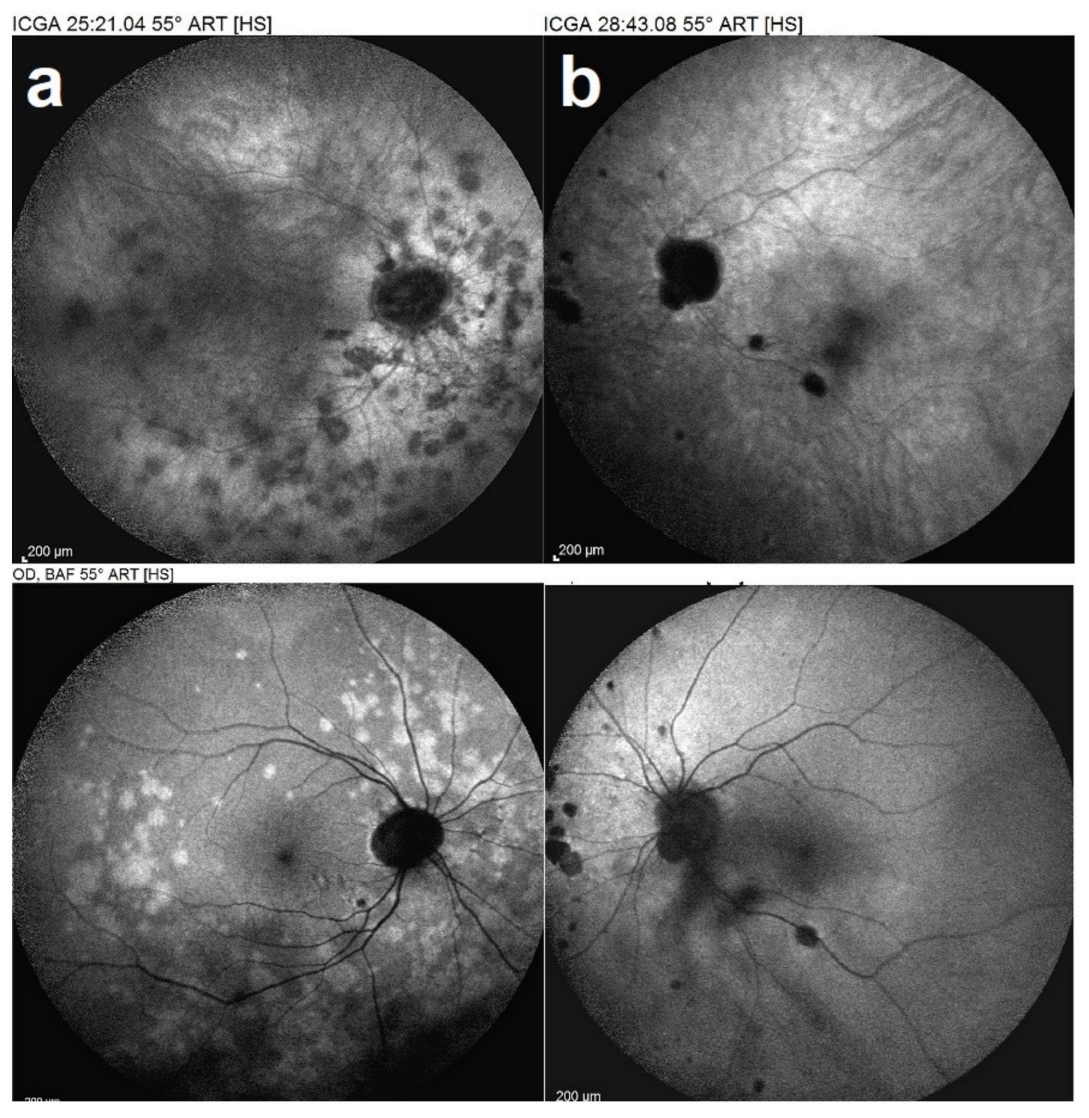
Idiopathic multifocal choroiditis, ICGA (top) and BL-FAF (**bottom**) findings. First episode of the right eye (**a**) in a period when left eye (**b**) showed no activity. ICGA OD (a **top**) showed characteristic hypofluorescent dots corresponding to the hyperautofluorescent dots in BL-FAF (a **bottom**), sign of activity.

**Figure 14:**
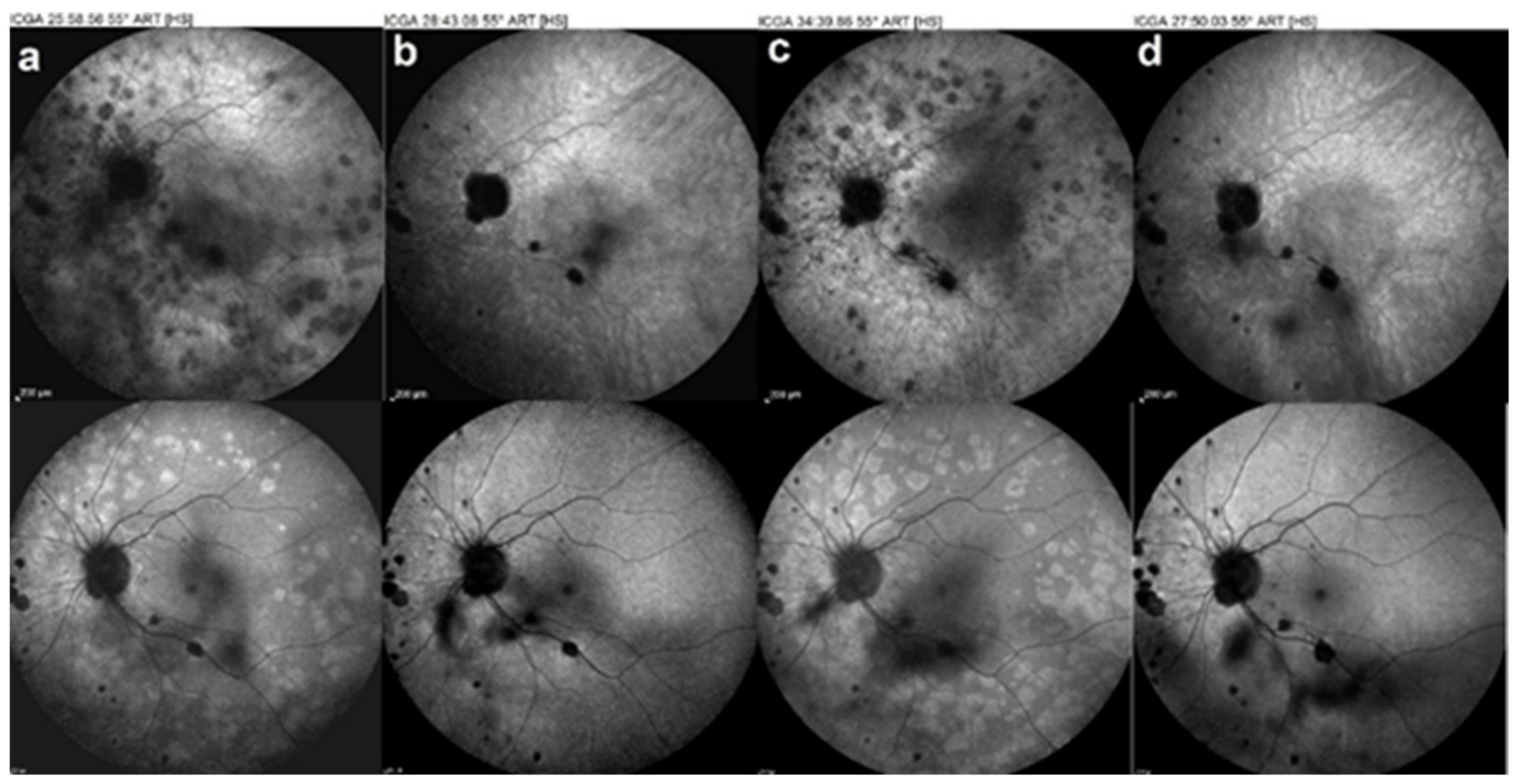
Idiopathic multifocal choroiditis case, follow-up of recurrences. ICGA (**top** images), BL-FAF (**bottom** images). Recurrences of the left eye as the patient refused the systemic treatment (**a**,**c**) (**b**,**d**) images after treatment.

**Figure 15:**
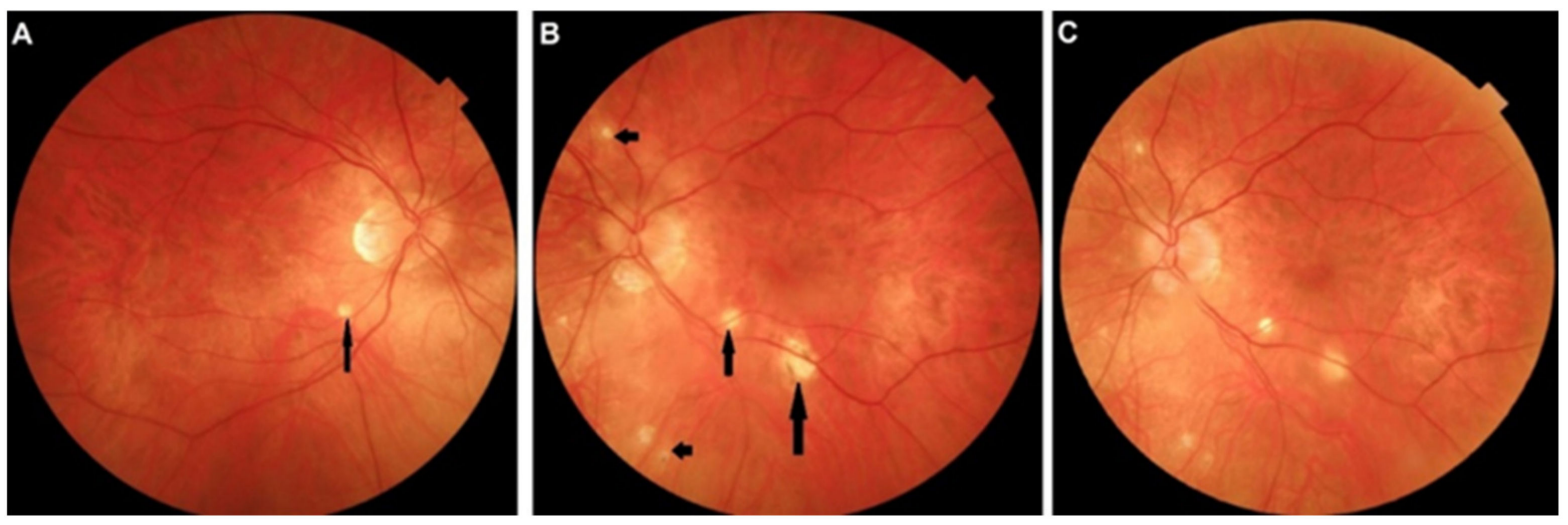
Idiopathic multifocal choroiditis. (**A**,**B**) Fundus aspect in last control, after 11 years of follow up. Scar lesions are outside the arcades of posterior pole (arrows). In (**C**), the fundus of the left eye 3 years before. Comparing (**B**) with (**C**), there are no additional scars for the last 3 years under systemic treatment.

Serpiginous choroiditis lies at the most severe end of the spectrum of the PICCPs [1,61,70]. It is a bilateral choriocapillaritis involving larger choriocapillaris vessels that progresses relentlessly in a creeping fashion if not treated rapidly and vigorously. At presentation, an IGRA test should be performed to rule out tuberculosis related SC, in which case additional anti-tuberculous antibiotics should be given in addition to immunosuppressive agents [77]. In the absence of immunosuppressive treatment, the condition will evolve and cause irreversible chorioretinal atrophy and severe functional impairment. Patients present with vision loss, photopsias and scotomas. No more than mild anterior chamber inflammation and faint vitritis can sometimes be noted. Fundus shows yellow-whitish serpentine, bilateral, although asymmetric lesions often starting from the optic disc and avoiding involving the central macula for some time, explaining the retained visual acuity until later in disease (Figure 4).

Multimodal imaging is not essential for the diagnosis, as fundus lesions are relatively pathognomonic, but allows us to better understand the clinicopathology of lesions and is extremely useful to monitor disease progression and response to treatment. ICGA clearly delineates the limits of the lesions that appear hypofluorescent throughout the angiographic sequence and account for both atrophic and active choriocapillaris non-perfusion. In addition, ICGA can show adjacent to the hypofluorescent areas, hyperfluorescent halos that indicate zones of progression of disease (Figure 4). FAF imaging depends on the stage of lesions. Lesion borders can show hyperautofluorescence as choriocapillaris and RPE are still present, but there is loss of photoreceptor outer segments while the central atrophic areas of lesions are hypoautofluorescent, as choriocapillaris and RPE are lost [8,78]. SD-OCT shows full-thickness absence of retinochoroidal layers in atrophic areas in the center of lesions and defect and damage to the photoreceptor outer segment layer at the border of lesions as well as presence of retinal oedema corresponding to the ICGA halo around atrophic lesion (Figure 4). OCT-A presents the classic choriocapillary drop-out, but images are limited to the posterior pole and cannot image more peripheral lesions. As for other PICCPs, OCT-A is helpful to detect ICNVs and monitor anti-VEGF treatment. FA reveals early hypofluorescent areas with late hyperfluorescence characteristics of active lesions. Scars appear hyperfluorescent during the whole angiographic sequence due to the window effect. VF shows extended scotomata, corresponding to active lesions and scars of the fundus.

**Scheme 4:**
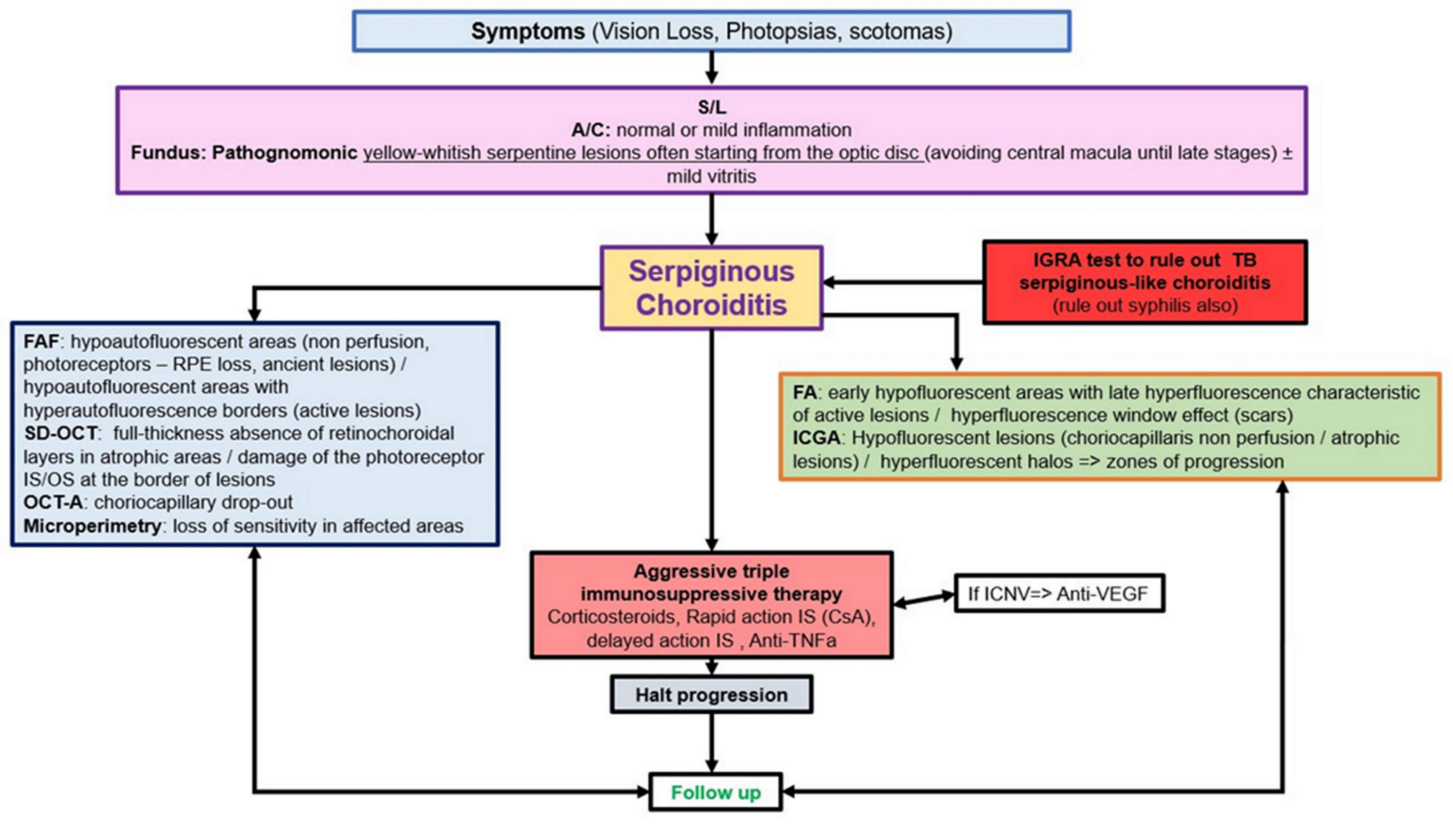
Appraisal of serpiginous choroiditis: decision tree in clinical practice. S/L: slit lamp. A/C: anterior chamber. IGRA: Interferon Gamma release assays. FAF: autofluorescence. SD-OCT: spectral domain optical coherence tomography. OCT-A: OCT-angiography. VF: visual fields. FA: fluoresceine angiography. ICGA: indocyanine green angiography. TB: tuberculosis. MFC: multifocal. APMPPE: acute posterior multifocal posterior placoid epitheliopathy. CNS: central neural system. Anti-VEGF: anti-vascular endothelial growth factor. ICNV: inflammatory choroidal neovascularization. IS: immunosuppressors. CsA: cyclosporine. Anti-TNFa: anti-tumor necrosis factor-a.

Prognosis is poor unless vigorous triple immunosuppressive therapy, as explained hereabove, is initiated early. Many reports put forward the importance of aggressive therapy [33,79,80,81]. Although the macula is spared in early disease, when lesions finally affect the fovea, vision is severely impaired. In order to halt the progression of the disease, anti-TNF-α agents or other biologicals [82] have to be given on top of triple immunosuppression, as long as the IGRA test is negative [77]. Appraisal of SC is schematized on Scheme 4.

#### Illustrative SC Case

A 71-year-old man complained of subjective scotomas in his left eye. His best corrected visual acuity (BCVA) was 1.0 OD and 0.8 OS. There was no anterior chamber inflammation and no vitritis. Fundus examination showed a macular yellow serpiginous atrophic lesion on the left side, while the right fundus was within normal limits.

FA showed a serpiginoïd hyperfluorescent lesion in the macula due to window effect of the atrophic fundus lesion. ICGA showed hypofluorescent areas OS corresponding to the fundus lesions and to the FA hyperfluorescence, but hypofluorescence was a little more widespread as it included both the atrophic areas and new areas of choriocapillaris hypo-non-perfusion. On the right side, ICGA showed occult hypofluorescent areas indicating choriocapillaris hypo-perfusion that had not yet caused atrophy with minimal lesions on OCT and survival of photoreceptor outer segments. Therefore, BL-FAF was normal.

**Figure 16:**
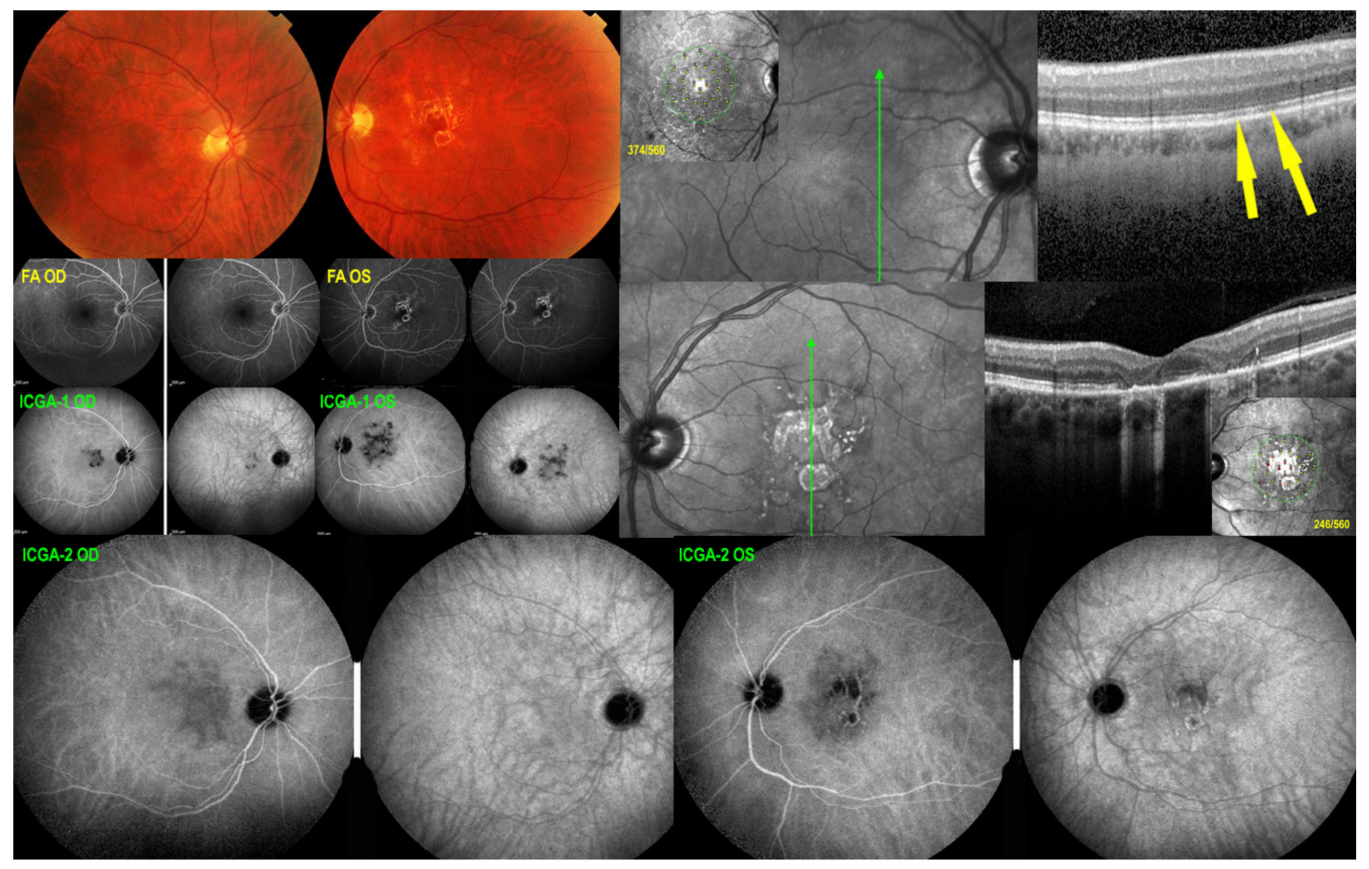
Serpiginous choroiditis. SC at first presentation. Fundus (**top left**) shows a normal posterior pole OD and a central chorioretinal scar OS. SD-OCT OD (**top right**) shows only faint IS/OS interruption (yellow arrows) with a slightly diminished retinal sensitivity (insert: microperimetry value 374/560, see Figure 19). SD-OCT OS (**middle right**) shows numerous zones of damage to the photoreceptor outer segments and total chorioretinal atrophic areas with markedly diminished retinal sensitivity (insert: microperimetry value 246/560, see Figure 19). FA OD appears as normal, and FA OS shows the chorioretinal scar (hyperfluorescent window effect). ICGA-1 OD shows occult hypofluorescent areas of choriocapillaris non-perfusion while ICGA-1 OS shows hypofluorescent areas that extend beyond the chorioretinal atrophic areas indicating additional occult non perfusion. After 3 sub-Tenon’s injection bilaterally, ICGA-2 OD shows decrease of hypofluorescence indicating reperfusion especially on the late frame (**right**). Similarly, ICGA-2 OS shows decrease of hypofluorescence in the areas around the central chorioretinal atrophic area indicating reperfusion of these areas.

The diagnosis of serpiginous choroiditis was retained. An IGRA test showed absence of contact with the tuberculous bacillus. Three bilateral sub-Tenon’s injections were performed at 6 weeks’ intervals which decreased ICGA hypofluorescence on both sides (Figure 16).

**Figure 17:**
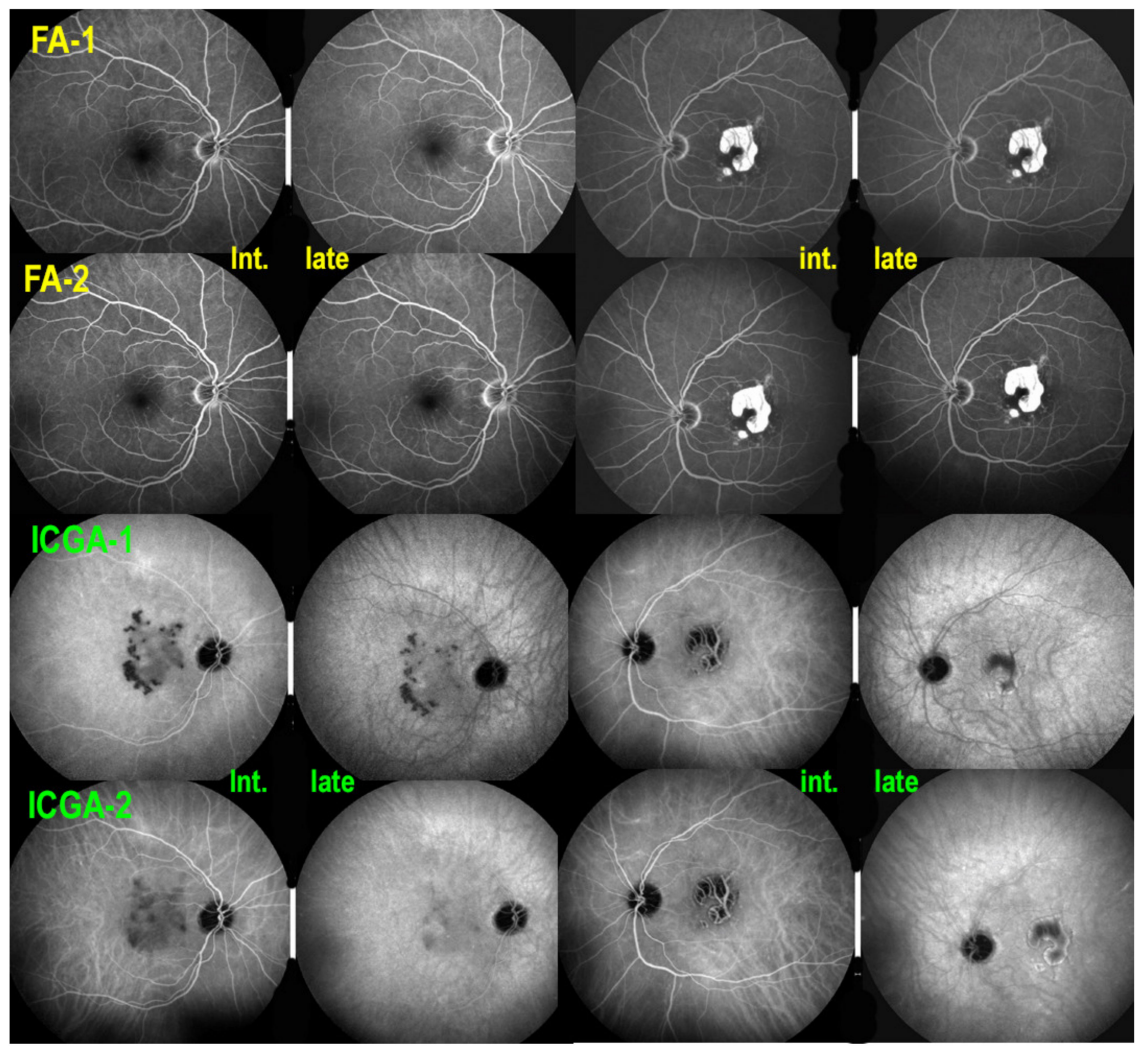
Serpiginous choroiditis. Recurrence of ICGA hypofluorescence 9 months after first presentation despite sub-Tenon’s injections of triamcinolone acetonide (40 mg). Initiation of triple immunosuppression (IS) is decided. FA-1 ODS before introduction of IS (top 4 frames) did not show occult increase of non-perfusion detected in both eyes by ICGA hypofluorescence (ICGA-1). After the introduction of IS, there was no change on FA (FA-2), while there was a marked reduction of hypofluorescence on ICGA (ICGA-2) especially on late frames, except for the irreversible chorioretinal atrophy OS.

**Figure 18:**
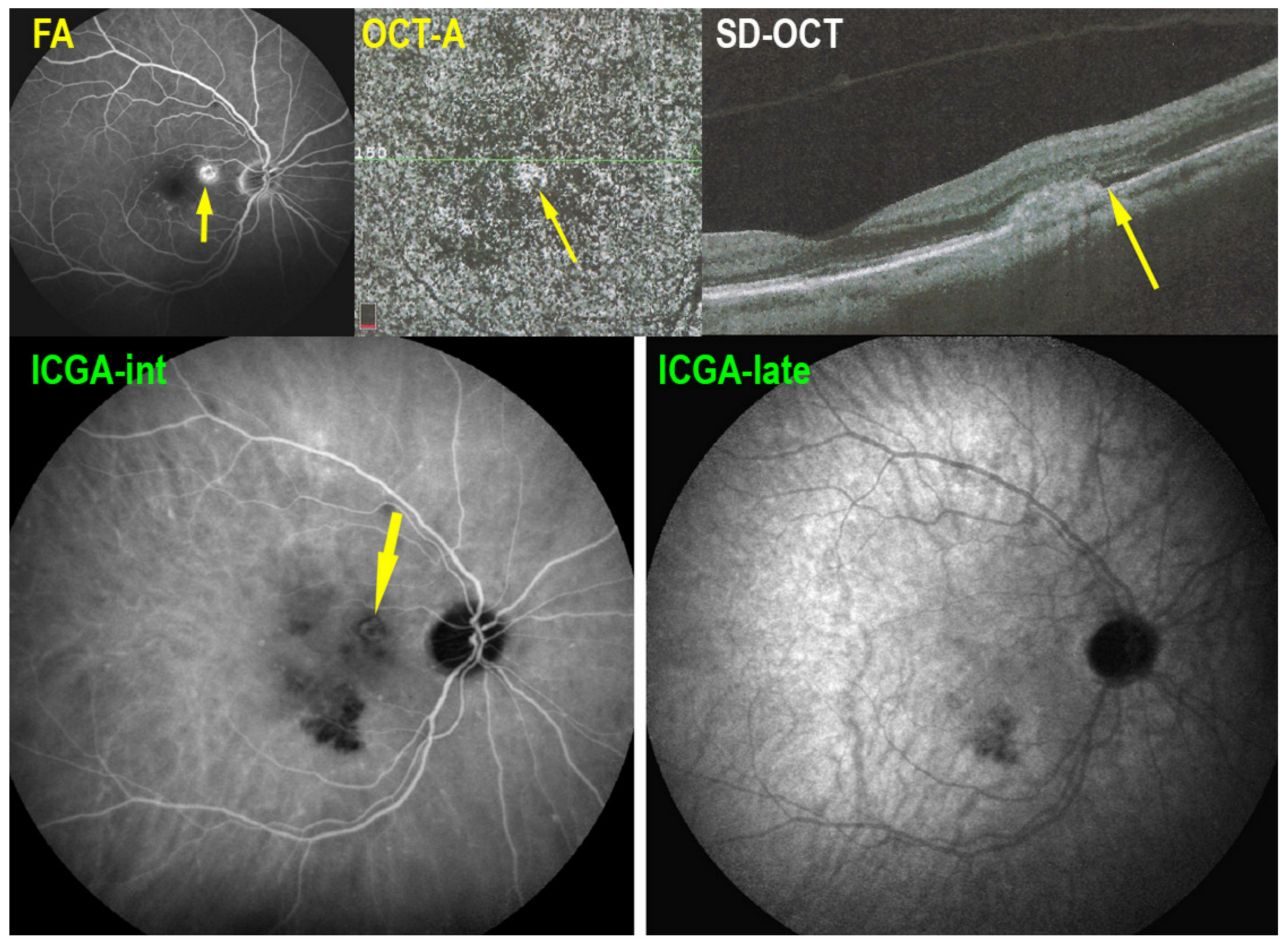
Serpiginous choroiditis: CNVs OD. Seven years after introduction of IS, hypofluorescence is still kept under control, especially in ICGA late frames (ICGA-late). However, parafoveolar CNVs were detected: yellow arrow on FA (late hyperfluorescent spot), OCT-A (presence of signal in en face OCT-A of the choriocapillaris), SD-OCT (hyperreflective lesion with fluid) and ICGA (hyperfluorescent ring surrounded by hypofluorescent halo), which responded well to one intravitreal injection of anti—VEGF agent (not shown).

However, ICGA hypofluorescence increased again bilaterally 9 months later (Figure 17). Therefore, triple immunosuppression including corticosteroids, cyclosporine (CsA/(Sandimmun^®^, 4.5 mg/kg) and mycophenolic acid (Myfortic^®^, twice 720 mg/day) caused again some regression of ICGA hypofluorescent lesions and stabilized the situation (Figure 17). Corticosteroids were tapered and discontinued after 3 months. Cyclosporine dosage was decreased from 4.5 mg/kg, but could never be stopped with fluctuating dosages between 2 and 4 mg/kg, and Myfortic^®^ (1440 per day) was maintained with a stable situation during a follow-up of 7 years (Figure 18).

**Figure 19:**
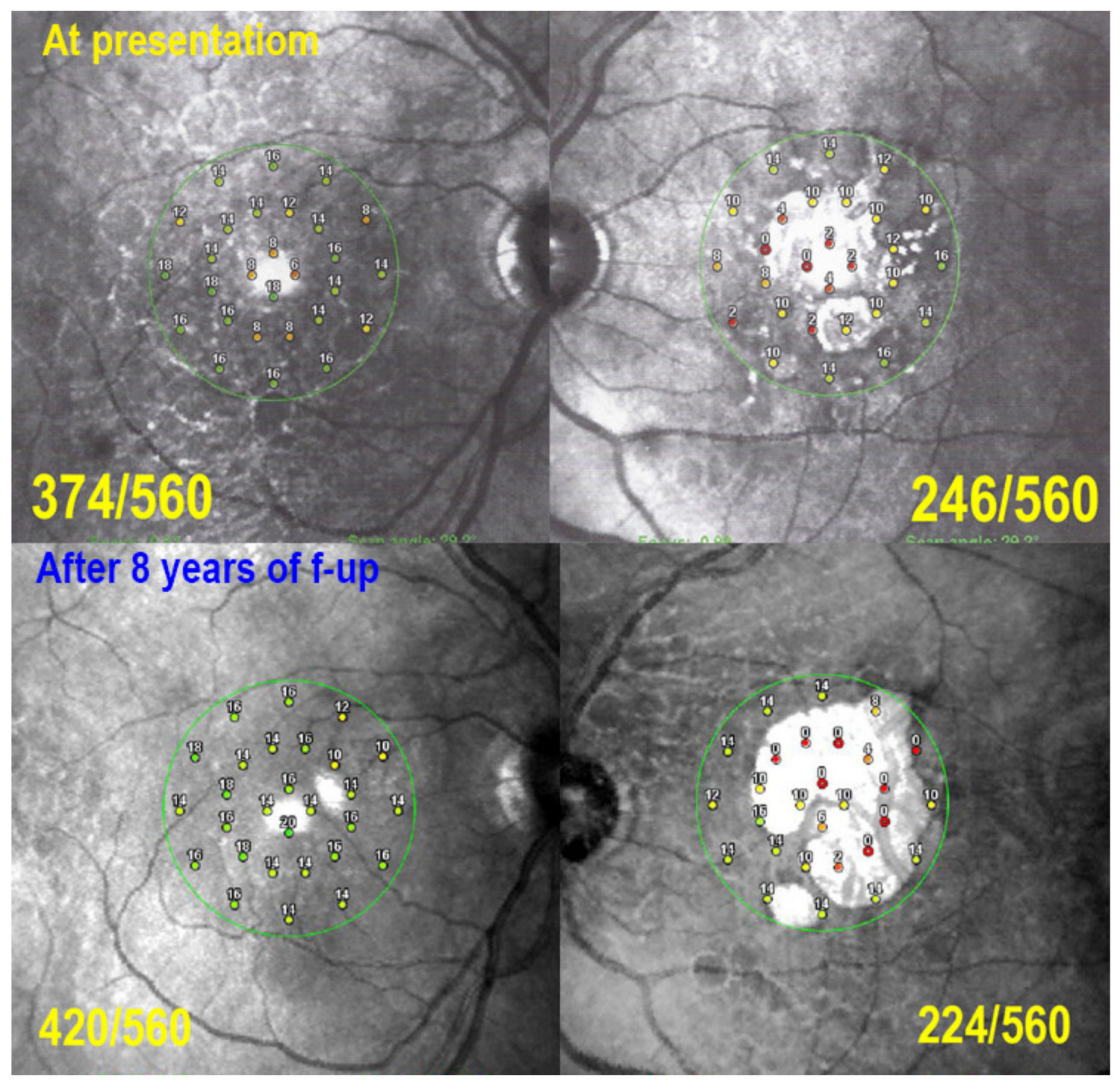
Serpiginous choroiditis: microperimetry follow-up. At presentation, there is a decrease of retinal sensitivity OD shown by microperimetry (374/560, **top left**), due to occult choriocapillaris hypo-perfusion seen on ICGA (see Figure 16). On the left side (**top right**), there is a substantial decrease of retinal sensitivity mainly due to chorioretinal atrophy (246/560, top right, see Figure 16). After 8 years of follow-up, under IS retinal sensitivity recovered OD (420/560, **bottom left**) and only slightly decreased OS (224/560, **bottom right**), indicating efficiency of prolonged IS.

CNVs developed OD after 7 years, successfully treated with two intravitreal injections of aflibercept (Eylea^®^). Six months later CNVs in OS developed, also successfully treated with one aflibercept intravitreal injection (not shown). Maintained vision and microperimetry values as well as decreased hypofluorescence was achieved after 8 years of follow-up under immunosuppression (Figure 19).

### 5.5. Mixed-Intermediary/Overlapping and Non-Classifiable Forms of Choriocapillaritis

**Figure 20:**
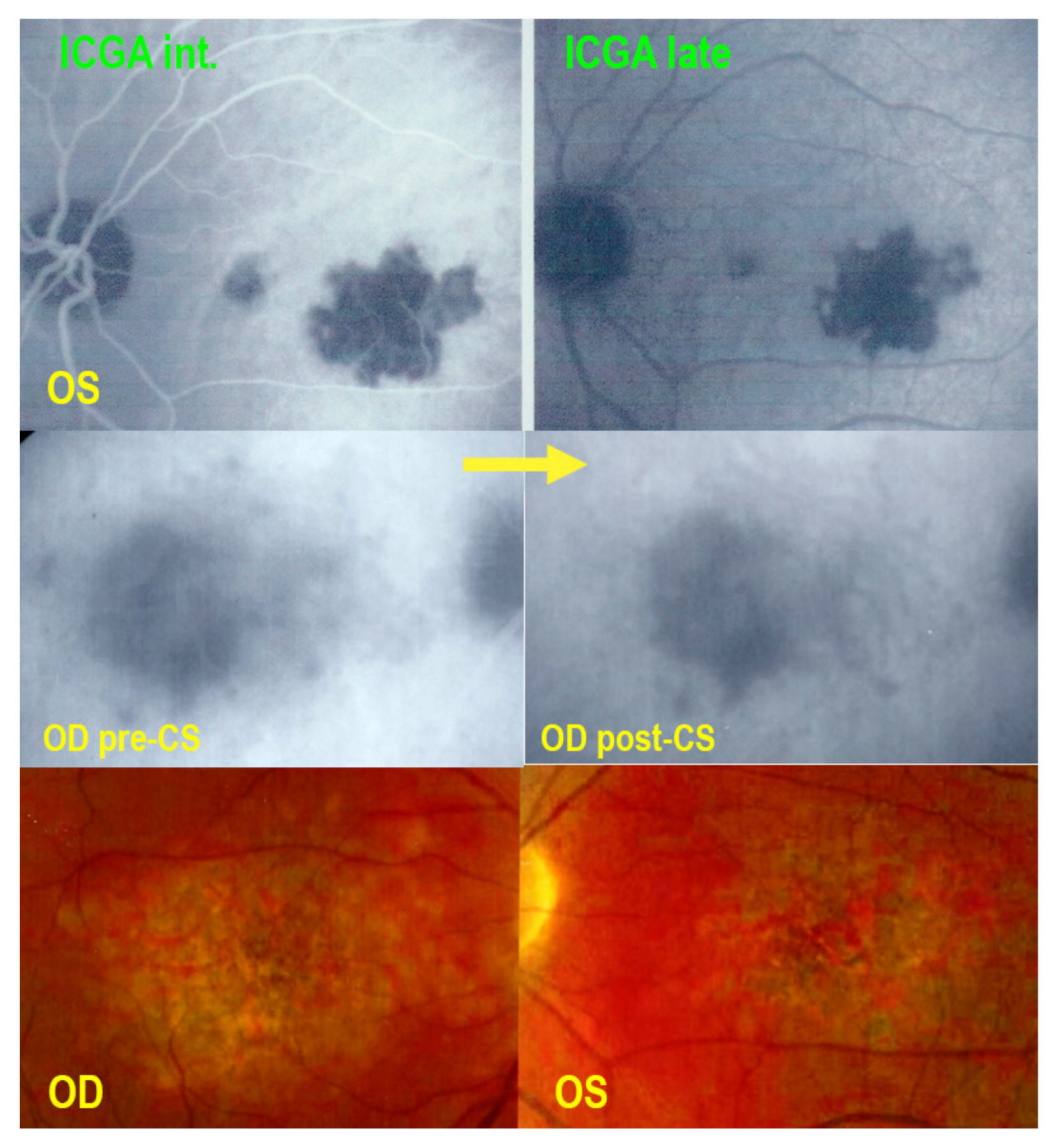
Non-classifiable case of choriocapillaritis. A middle-aged man was diagnosed as “unilateral APMPPE” OS (**Top** two frames) with a severe drop of vision after a flu-like episode. The doctors refrained from immunosuppressive treatment as “APMPPE is a self-limited disease” but macular atrophy ensued (**bottom** two frames). His right eye underwent the same evolution, less than a month later and the patient consulted another hospital where again nothing was undertaken as “APMPPE is a self-limited disease”. We saw the patient a few weeks later with macular atrophy OS and partial atrophy with choriocapillaris non-perfusion on ICGA OD (**middle left** picture- pre-CS) Systemic corticosteroid therapy was started, allowing to recover some areas of non-perfusion (**middle right**-post CS), but with a final low visual acuity. The first ICGA (**top** two frames) clearly showed choriocapillaris disease, which could, however, not be classified into one of the known entities.

Beside the four phenotypically well-determined entities exposed hereabove, many atypical cases of choriocapillaritis can develop. Mixed forms have been described associating the characteristics of two different choriocapillaritis phenotypes such as APMPPE and SC and was called “relentless placoid chorioretinitis” or “Ampiginous choroiditis”, conditions that combined the lesions of APMPPE and the evolution of SC [83,84]. Moreover, patients have been described whom presented as one entity such as MEWDS and subsequently evolved into MFC [7] or that first presented with a choriocapillaritis, MFC and later developed into a primary photoreceptoritis, AZOOR [38]. Many more reports of the occurrence of diverse PICCP conditions in the same patient have been published [85]. Finally, some cases cannot be classified precisely, as illustrated in Figure 20.

However, once choriocapillaritis has been established, close follow-up by multimodal imaging of all inflammatory choriocapillaropathies is mandatory and empiric corticosteroid and/or immunosuppressive therapy should be introduced in those cases with a deleterious functional and imaging evolution in order to avoid irreversible damage as shown on Figure 20.

## 6. Conclusions

Diagnosis and follow-up of PICCPs have become more performing and their clinicopathology have become better understood since the availability of multimodal imaging and especially since imaging access to the choroid has become possible through ICGA. The pathological process in all these conditions has now been identified, and the common denominator is inflammatory choriocapillaris perfusion disorders of various importance. These conditions with a well-defined pathophysiology should no more be classified with other unrelated diseases in the inappropriate and outdated terminology of white dot syndromes.

## Figures and Tables

**Figure 1:**
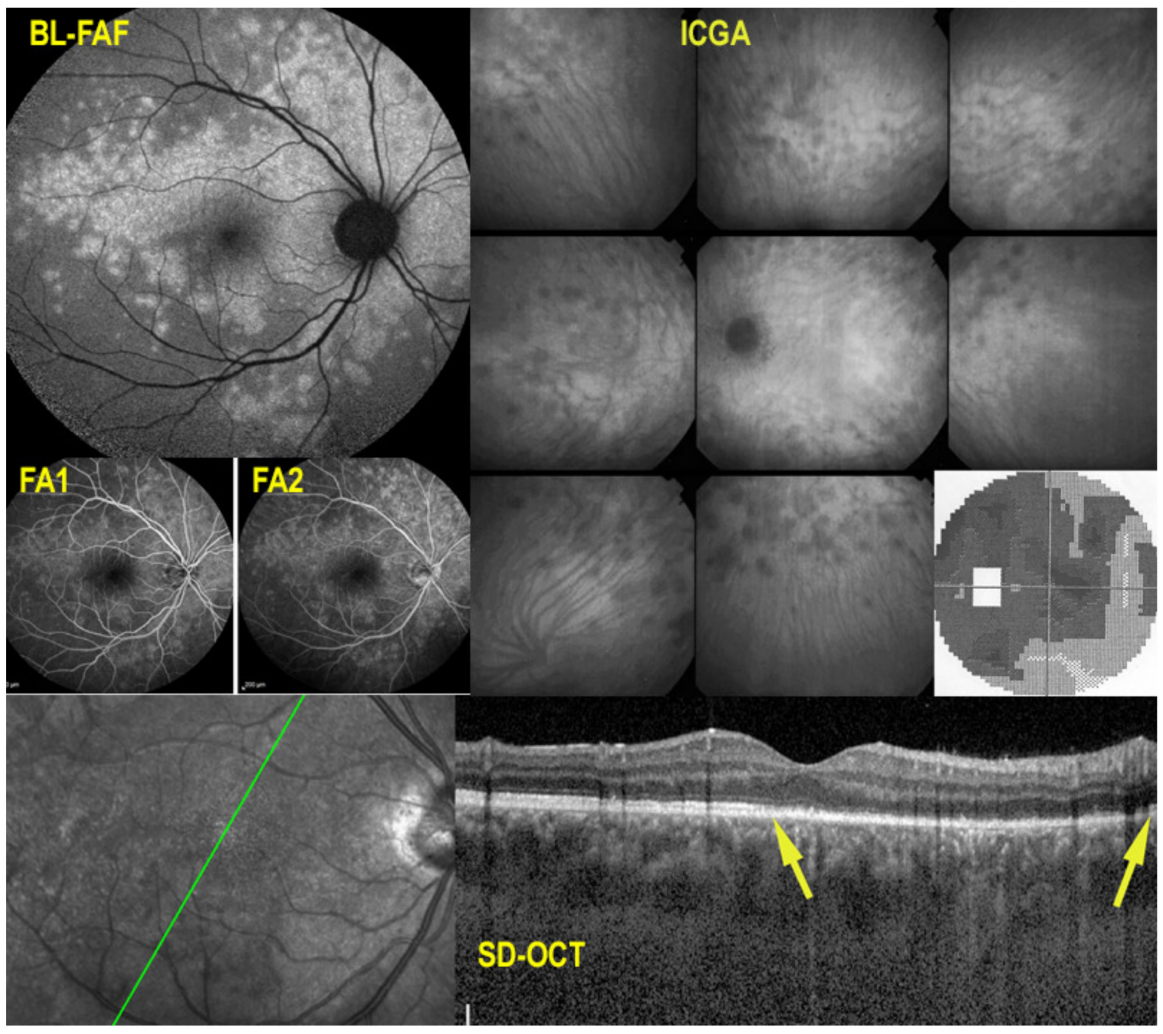
Multimodal imaging signs in MEWDS. BL-FAF (**top left**) showing geographic and confluent areas of hyperautofluorescence indicating active lesions. ICGA (**top right**, different patient) showing patchy areas of hypofluorescence with peripapillary hypofluorescence causing severe visual field loss (insert). FA (**middle left**) faint hyperfluorescence on early (FA1) and late (FA2) frames. SD-OCT (**bottom**) showing loss of photoreceptor outer segments (between yellow arrows).

**Table 1:** Clinical and demographic data of PICCPs.

	MEWDS	APMPPE/AMIC	MFC/PIC	Serpiginous
Age (y)	20–50	20–40	20–50	30–50
Gender	F	F = M	F	F = M
Laterality	Unilateral *	Bilateral/asymmetric	Bilateral/asymmetric	Bilateral/asymmetric
Myopia	Tendency towards myopia	Not important	Myopia is usual	Not important

* Very rare bilateral cases. MEWDS: Multifocal evanescent white dot syndrome. APMPPE: Acute posterior multifocal placoid pigment epitheliopathy. AMIC: Acute multifocal ischaemic choriocapillaritis. MFC/PIC: multifocal choroiditis/punctate inner choroiditis F: female/m: male Y: years.

**Table 3:** Anti-TNF-α agents’ main features.

	Mechanism of Action	Dose	Side Effects
**Infliximab** [35]	Chimeric monoclonal antibody, bound to both transmembrane and soluble form of TNF-a. Kills cells that express TNF-a	iv 5–20 mg/kg/day Loading dose at 0, 2, 4 weeks then every 6–10 weeks	Reactivation of infections Lupus like syndrome Malignancy (lymphoproliferative disease)
**Adalimumab** [36]	Human monoclonal antibody, same as infliximab	SC 40 mg every 2 weeks (in severe cases interval can decrease to 7–10 days [40])	Headache, nausea, stomach-ache Secondary malignancy Demyelinating disorder

## Data Availability

Please refer to corresponding authors.
